# Postoperative functional rehabilitation for cervical spondylotic myelopathy: An integrative review guided by Orem’s self-care deficit theory

**DOI:** 10.1016/j.ijnsa.2026.100696

**Published:** 2026-10-01

**Authors:** Xiaoping Wang, Xuerong Zhang, Qing Zhao, Can Liu, Yanling He, Meifang Li

**Affiliations:** Department of Orthopedics, Zhongshan Xiaolan People’s Hospital (The Fifth People’s Hospital of Zhongshan), Xiaolan Clinical Institute of Shantou University Medical College, No. 65, Jucheng Road, Zhongshan, 528415, China

**Keywords:** Cervical spondylotic myelopathy, Postoperative rehabilitation, Nursing care, Self-care deficit nursing theory, Kinesiophobia, Functional exercise, Integrative review

## Abstract

**Background:**

Cervical spondylotic myelopathy is the predominant cause of spinal cord dysfunction in adults, for which surgical decompression remains the mainstay of treatment. Surgical success alone does not guarantee functional restoration, and postoperative rehabilitation is a critical determinant of long-term outcome. A comprehensive, theory-grounded nursing framework to guide rehabilitation practice for this population has not been available.

**Objectives:**

To synthesise the current evidence on postoperative rehabilitation nursing for patients with cervical spondylotic myelopathy, to develop a theory-informed nursing framework anchored in Orem’s Self-Care Deficit Nursing Theory, and to identify research gaps and priorities.

**Design:**

Integrative review following the methodology of Whittemore and Knafl, with reporting guided by the Preferred Reporting Items for Systematic Reviews and Meta-Analyses 2020 statement where applicable.

**Information sources:**

PubMed/MEDLINE, CINAHL Complete, Web of Science Core Collection, Embase and OpenAlex, together with reference-list screening, searched for reports published between January 2014 and January 2025.

**Methods:**

Two reviewers independently screened records, extracted data and appraised methodological quality using instruments appropriate to each study design. A theory-driven narrative synthesis with thematic analysis was performed, and the findings were mapped onto the constructs of Orem’s theory.

**Results:**

Fifty-one studies met the inclusion criteria. Four synthesis domains were identified: risk stratification and acute-phase management; phased functional exercise rehabilitation; pain management and psychobehavioural support; and long-term follow-up and telerehabilitation. Psychological barriers were prominent; a single-centre study reported a kinesiophobia prevalence of 75.4% after surgery, although this estimate derives from limited evidence and requires confirmation in larger, multi-centre cohorts. A Rehabilitation Nursing System Selection Framework was developed to operationalise the constructs of Orem’s theory as a conceptual basis for individualising postoperative nursing care.

**Conclusion:**

Effective postoperative rehabilitation after surgery for cervical spondylotic myelopathy requires concurrent attention to physical and psychological dimensions. The kinesiophobia prevalence reported in individual studies suggests that screening for psychological barriers may help identify patients who could benefit from additional psychological or behavioural support alongside physical rehabilitation. The proposed framework provides a foundation for developing standardised nursing protocols and for advancing intervention research in this field.

**Registration:**

Registered retrospectively with the Open Science Framework Registries (https://osf.io/4jh7q) on 12 December 2025.

**What is already known**

• Surgical decompression constitutes the standard of care for moderate-to-severe cervical spondylotic myelopathy.
• Postoperative rehabilitation influences functional outcomes, though the evidence base remains fragmented across disparate studies.
• Fear of movement has been implicated in rehabilitation outcomes across musculoskeletal conditions, but its role after surgery for cervical spondylotic myelopathy is under-investigated.

**What this paper adds**

• Individual studies report a high prevalence of kinesiophobia (up to 75.4%) and of preoperative psychological distress (31%) after surgery for cervical spondylotic myelopathy, although these estimates derive from limited, predominantly single-centre evidence.
• Orem's Self-Care Deficit Nursing Theory can be operationalised into a framework that matches nursing system selection to the level of self-care deficit across the postoperative pathway.
• Integrating psychobehavioural screening into nursing assessment enables differentiation between physical limitations and deficits in self-care agency, which call for different nursing responses.

## 1 Introduction

Cervical spondylotic myelopathy stands as the most prevalent cause of spinal cord dysfunction in adults globally, precipitating progressive neurological deterioration, functional impairment, and diminished quality of life (Nouri et al., 2015; Fehlings et al., 2017). Epidemiological data reveal an age-dependent increase in prevalence coupled with significant genetic predisposition (Nouri et al., 2015). Surgical decompression constitutes the standard treatment for moderate-to-severe myelopathy, with large-scale prospective studies demonstrating that 76% of patients achieve minimal clinically important difference at one year (Fehlings et al., 2013). Surgical intervention alone, however, is often insufficient to ensure optimal functional recovery. Postoperative rehabilitation assumes an essential role in bridging the chasm between surgical decompression and functional restoration, with nursing care forming an integral component of this rehabilitative trajectory.

Perioperative rehabilitation for cervical spondylotic myelopathy spans a continuum from preoperative optimisation (prehabilitation) through acute inpatient recovery to community-based reintegration. Prehabilitation seeks to improve physiological reserve and address modifiable risk factors before surgery; the acute phase focuses on safe mobilisation, complication prevention, and restoration of basic self-care; and the community phase emphasises functional consolidation, self-management, and monitoring for late deterioration. Evidence across these phases is uneven: most high-quality data concern surgical decompression and short-term neurological outcomes, whereas structured rehabilitation, and particularly the nursing contribution to it, remains comparatively under-investigated (Badran et al., 2018; Boerger et al., 2022; Smith et al., 2025). Whereas physiotherapy and rehabilitation medicine have well-defined roles in motor recovery, nursing uniquely integrates physical, educational, and psychobehavioural support across the whole perioperative pathway, positioning nurses to coordinate care and to address barriers such as fear of movement and psychological distress.

Despite the expanding evidence base on surgical techniques and outcomes in cervical spondylotic myelopathy, a critical gap persists in the nursing literature: no comprehensive, theory-grounded framework currently exists to inform rehabilitation nursing practice for patients with cervical spondylotic myelopathy. Recent systematic reviews have underscored the paucity of evidence specifically examining rehabilitation following degenerative cervical myelopathy surgery (Badran et al., 2018; Smith et al., 2025), while simultaneously identifying promising directions for peri-operative rehabilitation development (Boerger et al., 2022). The existing literature remains fragmented, with individual studies addressing discrete interventions without integration into a unified conceptual model. Clinical recommendations frequently lack theoretical grounding, and nursing’s distinctive contribution relative to other rehabilitation disciplines remains inadequately delineated. This lacuna assumes particular significance in light of emerging evidence that psychological factors, including kinesiophobia (fear of movement) and preoperative distress, exert substantial influence on rehabilitation outcomes, factors that fall squarely within nursing’s scope of practice.

This integrative review was undertaken to: (1) synthesize the current evidence on postoperative rehabilitation nursing and functional exercise for patients with cervical spondylotic myelopathy; (2) develop a theory-grounded, conceptual nursing framework utilizing Orem’s Self-Care Deficit Nursing Theory; and (3) delineate research gaps and priorities for future nursing scholarship in this domain.

## 2 Conceptual framework

This review employs Orem's Self-Care Deficit Nursing Theory as an a priori analytical framework to structure evidence synthesis. The following sections operationalize Orem’s core constructs for the postoperative rehabilitation context.

### 2.1 Overview of Orem's self-care deficit nursing theory

Orem's Self-Care Deficit Nursing Theory encompasses three interrelated theoretical constructs: the Theory of Self-Care, the Theory of Self-Care Deficit, and the Theory of Nursing Systems (Orem, 2001). The theory posits that individuals possess an inherent capacity for self-care, defined as deliberate, purposive actions undertaken to maintain health and well-being. When self-care demands exceed an individual's self-care agency, a self-care deficit emerges, establishing the legitimate need for nursing intervention. The theory delineates three nursing systems: wholly compensatory (wherein the nurse executes all self-care actions), partly compensatory (wherein the nurse and patient share responsibility), and supportive-educative (wherein the patient performs self-care with nursing guidance and instruction).

### 2.2 Operationalizing core constructs for cervical spondylotic myelopathy rehabilitation

Self-care agency denotes the capability enabling individuals to engage in self-care activities. For postoperative patients with cervical spondylotic myelopathy, we operationalize self-care agency across three domains: cognitive-knowledge (comprehension of precautions and the rehabilitation trajectory), motivational-volitional (psychological readiness and self-efficacy beliefs), and physiological-functional (physical capacity for self-care activities). We emphasise that this three-domain structure is an author-developed operationalisation proposed for the postoperative rehabilitation context, rather than a taxonomy drawn directly from Orem's original theory.

Therapeutic self-care demand encompasses the totality of self-care actions required to meet self-care requisites. For postoperative patients with cervical spondylotic myelopathy, these demands include: universal requisites (maintaining fundamental physiological functions), developmental requisites (adapting to altered capabilities), and health deviation requisites (adhering to treatment protocols and monitoring for complications).

### 2.3 The rehabilitation nursing system selection framework

Grounded in Orem’s theoretical foundation, we propose a conceptual Rehabilitation Nursing System Selection Framework derived from this review (Fig. 1). This framework comprises four phases: (1) comprehensive assessment encompassing neurological, functional, and psychobehavioral status; (2) self-care deficit determination through systematic comparison of therapeutic demands against patient agency; (3) nursing system selection (wholly compensatory, partly compensatory, or supportive-educative); and (4) dynamic reassessment as patient status evolves along the recovery trajectory. This framework is intended to organise existing evidence and to guide future research; it is not a validated clinical decision tool and requires prospective empirical validation before clinical application. The application of this framework to the synthesized evidence is elaborated in the Results section.

**Fig. 1:**
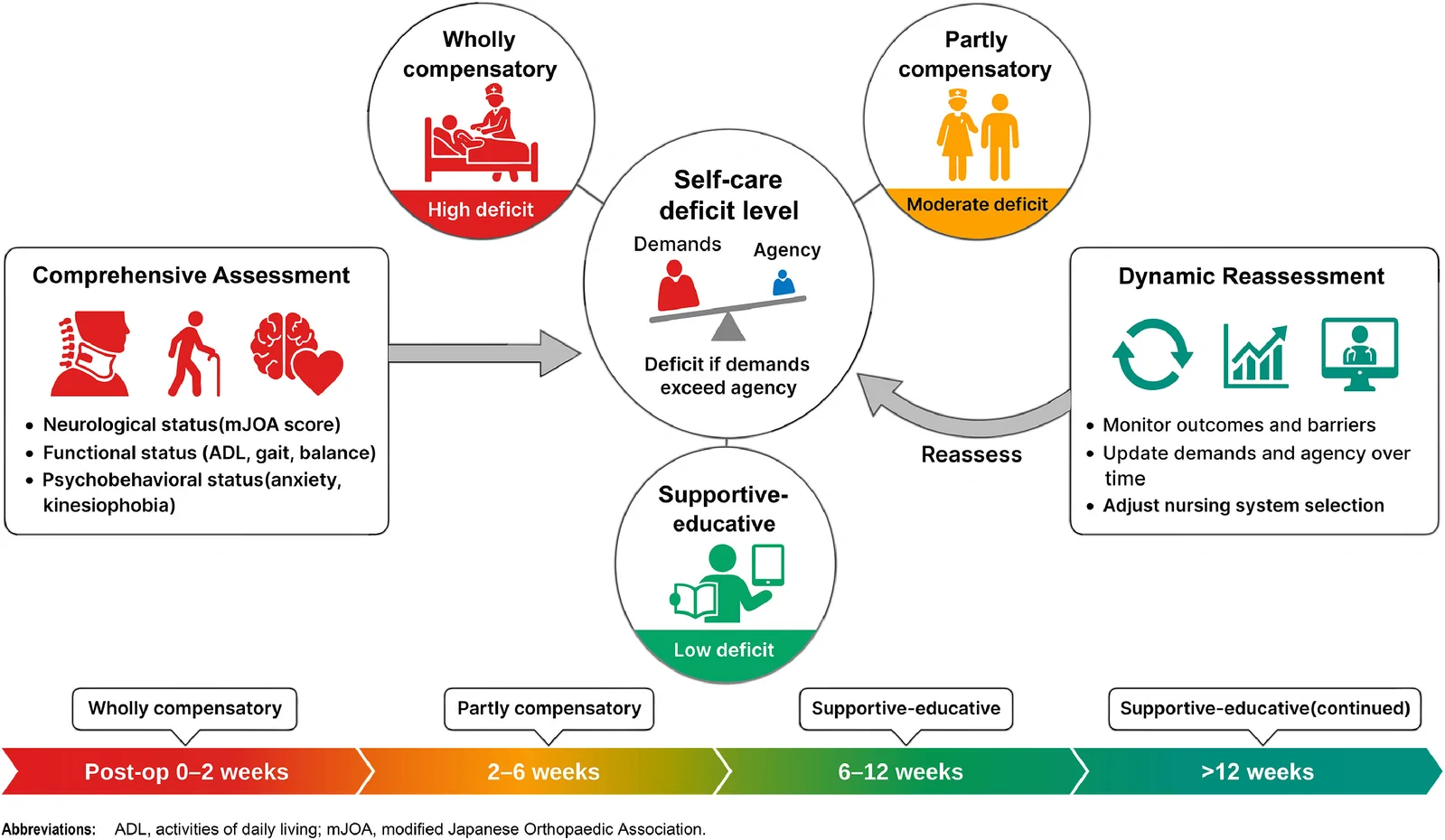
**The Rehabilitation Nursing System Selection Framework: Operationalizing Orem's Self-Care Deficit Theory**. This framework guides nurses through: (1) comprehensive assessment of neurological status (modified Japanese Orthopaedic Association score), functional status (activities of daily living, gait and balance), and psychobehavioral factors (anxiety/kinesiophobia); (2) determining self-care deficit by comparing therapeutic demands against patient self-care agency; (3) selecting the appropriate nursing system: wholly compensatory (acute phase), partly compensatory (rehabilitation phase), or supportive-educative (long-term follow-up); and (4) continuous reassessment for dynamic system transitions along the recovery trajectory. This Fig. was redrawn as vector artwork with the assistance of Claude (Anthropic); the authors reviewed and verified its content. Abbreviations: ADL, activities of daily living; mJOA, modified Japanese Orthopaedic Association.

## 3 Methods

### 3.1 Design

This study employed an integrative review design following the methodological framework proposed by Whittemore and Knafl (2005). An integrative approach was selected to enable the synthesis of empirical evidence derived from diverse methodological traditions, including quantitative, qualitative, and mixed-methods studies. Given the complexity of postoperative rehabilitation for cervical spondylotic myelopathy and the multifaceted nature of nursing interventions, this design allowed comprehensive integration of clinical, behavioral, and contextual dimensions relevant to nursing practice.

To enhance methodological transparency and rigor, the conduct and reporting of this review were guided, where applicable, by the Preferred Reporting Items for Systematic Reviews and Meta-Analyses (PRISMA) 2020 statement for systematic reviews.

### 3.2 Theoretical framework and analytical orientation

Orem’s Self-Care Deficit Nursing Theory was adopted as the overarching theoretical framework guiding this integrative review. The theory conceptualizes nursing as a response to deficits in individuals’ self-care agency relative to their therapeutic self-care demands, operationalized through three nursing systems: wholly compensatory, partly compensatory, and supportive–educative (Orem, 2001).

In this review, Orem’s theory functioned not merely as an interpretative lens but as an a priori analytical scaffold. Core theoretical constructs, including self-care agency, self-care deficit, therapeutic self-care demand, and nursing system selection, were used to inform data extraction, coding, and synthesis. This theory-guided orientation ensured that evidence synthesis remained explicitly anchored in nursing knowledge development rather than biomedical rehabilitation paradigms.

### 3.3 Search strategy

A systematic literature search was executed in February 2025 across five electronic databases: PubMed/MEDLINE, CINAHL Complete, Web of Science Core Collection, Embase, and OpenAlex. An initial search had been run in November 2024 across the databases specified in the registered protocol; that search was superseded by the February 2025 search, which used the amended set of databases and an extended window and was executed in full rather than as an update, so that the review would rest on a single, fully documented search. Title, abstract and full-text screening were carried out afresh on its complete yield, and the records reported in Fig. 2 are those of the February 2025 search. The sequence, and the reasons for it, are set out in the protocol statement at the end of this section. Embase was included to ensure comprehensive coverage of European clinical literature and rehabilitation research that may not be fully indexed in other databases. OpenAlex was included to maximize retrieval comprehensiveness, as it indexes a broader range of interdisciplinary and open-access literature. This approach aligned with integrative review methodology, which seeks to incorporate diverse evidence sources (Whittemore and Knafl, 2005). The search encompassed publications from January 2014 to January 2025. Seminal studies published before 2014 were retained to inform the theoretical framework and provide foundational context for rehabilitation practices in cervical spondylotic myelopathy; these studies were not included in the total count of included studies but are cited for their theoretical and clinical significance. The search strategy combined Medical Subject Headings terms, Emtree terms, and free-text keywords. Search terms comprised: population terms ("cervical spondylotic myelopathy," "cervical myelopathy," "degenerative cervical myelopathy"), intervention terms ("surgery," "decompression," "postoperative"), and outcome terms ("rehabilitation," "nursing," "functional exercise", "nurse-led," "nursing intervention," "self-care," "patient education"). The complete search strategy for each database is provided in Supplementary Material S1. Reference lists of included studies underwent manual screening to identify additional relevant articles.

**Fig. 2:**
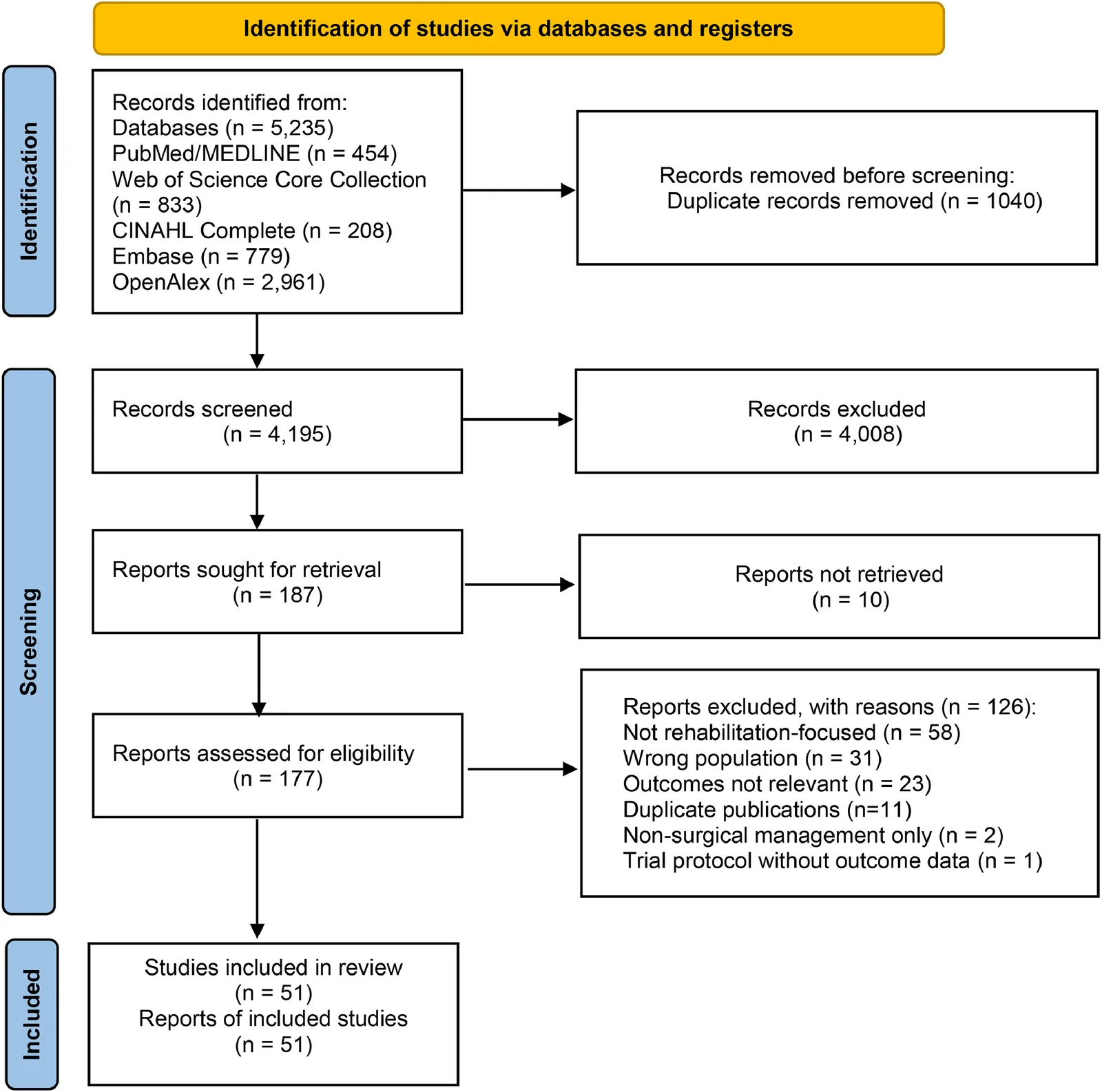
**PRISMA 2020 flow diagram.** Flow diagram illustrating the systematic study selection process from identification through screening, eligibility assessment, and final inclusion.

Protocol registration and departures from the registered record. The review protocol was registered with the Open Science Framework Registries (https://osf.io/4jh7q) on 12 December 2025. Registration was therefore retrospective: it followed both the execution of the search and the completion of the synthesis, and it was made at that point because PROSPERO does not accept reviews that have progressed to data extraction. The registered record reproduces the protocol as originally drafted for this review and was not updated before posting to reflect the procedures that had by then been applied. The registered record and the review as conducted therefore differ in six respects, which are set out below with the reason for each. None of the procedures reported here was adopted in response to the findings of an individual study, and none altered the review question or the outcomes of interest. Because registration followed the synthesis, it does not protect against selective reporting.

First, the databases searched were changed. The registered plan listed PubMed/MEDLINE, CINAHL Complete, Web of Science Core Collection and Google Scholar, together with reference-list screening. Google Scholar was replaced by OpenAlex, because Google Scholar does not return exportable, stably ordered result sets and so cannot support a reproducible and deduplicable search record, whereas OpenAlex returns structured, identifier-indexed records that can be queried and deduplicated reproducibly; Embase was added for coverage of European clinical and rehabilitation literature that is incompletely indexed elsewhere. Reference-list screening was retained as registered. Both changes were made when the search was re-run, and the re-run was executed in full rather than as an update to the earlier search. Second, the end of the search window was moved from October 2024 to January 2025, the start date of January 2014 being as registered. The initial search of November 2024 covered the period to October 2024; when the search was re-run in February 2025, the window was extended so that it ran to the month preceding execution. Third, language eligibility was widened from English only to English or Chinese, so that Chinese-language reports of postoperative rehabilitation nursing could be considered; in the event no Chinese-language report met the remaining criteria, and all 51 included studies are English-language publications, this widening did not alter the corpus. Fourth, design eligibility was broadened beyond randomized controlled trials, quasi-experimental studies, cohort studies, systematic reviews, clinical guidelines and expert consensus statements to admit cross-sectional studies, meta-analyses, measurement and validation studies, and narrative reviews, consistent with integrative-review methodology, which accommodates diverse forms of evidence provided that the role of each in the synthesis is made explicit (Whittemore and Knafl, 2005). Fifth, the population criterion was extended: the registered plan restricted eligibility to adults with cervical spondylotic myelopathy who had undergone surgical decompression, whereas the review as conducted additionally admitted a defined and separately labelled tier of adjacent-population evidence, described below, because several components of postoperative rehabilitation nursing had no evidence base specific to surgically managed cervical spondylotic myelopathy. This extension is confined to adjacent surgical and musculoskeletal populations. The registered exclusion of studies focused exclusively on non-surgical management was maintained unchanged. Sixth, and consequent on the fourth difference, two instruments were added to the registered appraisal set of JBI checklists, AMSTAR 2 and AGREE II, namely COSMIN for measurement and validation studies and SANRA for narrative reviews.

Two further differences from the registered record concern the strength of claims rather than method, and in both the claim was narrowed rather than widened during peer review. The registered objectives included identifying the psychobehavioural burden as a critical barrier to rehabilitation success; those findings are reported here as one component of the synthesis rather than as its central finding, because the prevalence estimates derive from limited, predominantly single-centre evidence. The registered anticipated output was a clinically actionable nursing system selection model; it is presented here as a conceptual framework requiring prospective empirical validation before use as a clinical decision tool. The registered plan, the procedure finally applied, and the rationale for every difference are set out in full in Supplementary Material S3 (Table S3.1).

### 3.4 Eligibility criteria

The criteria set out below are those applied to the final corpus. Where they differ from the registered record, the difference and its reason are reported in the protocol statement above and in Supplementary Material S3. The inclusion criteria are structured in two tiers, distinguishing evidence generated in the target population from evidence generated in clinically adjacent populations and used as transferable rather than direct evidence. The exclusion criteria are applied as registered.

#### 3.4.1 Inclusion criteria

Tier 1 (core evidence). Studies were included in the core tier if they satisfied all of the following: (1) Population: adults (≥ 18 years) with cervical spondylotic myelopathy or degenerative cervical myelopathy managed by surgical decompression, or cervical spine surgical cohorts in which myelopathy was an operative indication; (2) Focus: postoperative rehabilitation interventions, nursing care processes or protocols, functional exercise programmes or self-care support strategies, or prognostic, measurement, guideline or consensus evidence bearing directly on postoperative recovery and its assessment; (3) Outcomes: neurological recovery, functional independence, quality of life, pain, complications, psychological outcomes, or length of stay; (4) Design: randomized controlled trials, quasi-experimental studies, prospective or retrospective cohort studies, cross-sectional studies, systematic reviews and meta-analyses, clinical practice guidelines and consensus statements, measurement or validation studies, and narrative reviews.

Tier 2 (adjacent, transferable evidence). Studies conducted outside the surgically managed cervical myelopathy population were included only where all three of the following conditions were met: (i) the population was clinically adjacent, comprising mixed or non-cervical spinal surgical cohorts, spinal cord injury, or chronic neck pain; (ii) the study addressed a component of postoperative rehabilitation or nursing care that no Tier 1 study addressed; and (iii) its contribution could be stated as transferable rather than direct evidence. Studies of the non-surgical management of cervical myelopathy were not admitted under this tier, since they fall under exclusion criterion (1) below.

Tier 2 studies were appraised with the same design-appropriate instruments as Tier 1 studies, were labelled by evidence type in Table 1, and were not used as the sole basis for any practice-oriented statement; statements resting on them are identified as transferable in the Results. Of the 51 included studies, 42 (82.4%) met the Tier 1 criteria and 9 (17.6%) were admitted under Tier 2; the latter are marked in Table 1.

**Table 1:** Characteristics of included studies by synthesis domain (N = 51).

First author (Year)	Country	Design	Evidence type	n	Population/diagnosis	Procedure	Intervention/rehabilitation components	Timing/phase	Outcome measures	Key findings	**Appraisal tool**	**Score/rating**
**Domain 1: Risk stratification and acute-phase management (n = 15)**												
bb	Japan	Prospective cohort	Nursing	209	Adults undergoing spinal surgery (mixed spinal procedures)	Spinal surgery (various)	Institutional VTE-prophylaxis protocol: pneumatic sequential compression device + graduated compression stockings; peri-operative lower-limb ultrasonography	Peri-operative / acute	VTE incidence (DVT, PE) by ultrasonography; prophylaxis effectiveness	Peri-operative VTE ∼11% (9/209 pre-existing DVT [4.3%]; 14 new-onset post-op); routine ultrasonographic DVT screening deemed vital; mechano-prophylaxis effective	JBI (Cohort)	11/11 (High)
Cox et al., (2014)^b^	USA	Retrospective cohort	Nursing	2,846	Adults (≥18 y) admitted for spine surgery; pre-protocol vs post-protocol groups	Spine surgery (various)	Early multimodal VTE prophylaxis: sequential compression devices + subcutaneous heparin started preoperatively/same day + early mobilisation	Peri-operative / early post-op	VTE (DVT, PE) rates; epidural haematoma	Early multimodal prophylaxis reduced DVT (25→10) and PE without excess epidural haematoma; supports early chemoprophylaxis plus mechanical devices and early mobilisation	JBI (Cohort)	11/11 (High)
Nouri et al. (2015)	Canada	Narrative review	Contextual	–	Adults with degenerative cervical myelopathy (evidence synthesis)	Not applicable (narrative review)	Not applicable (review of epidemiology/genetics/pathogenesis)	Whole disease course (contextual)	Prevalence, aetiology, pathogenesis	Established DCM as the commonest cause of adult spinal-cord dysfunction; age-related prevalence and genetic predisposition; framed pathophysiology relevant to recovery	SANRA	8/12 (Moderate)
Nakashima et al., (2016)	Multicentre (AOSpine)	Prospective cohort	Prognostic	479	Adults with DCM stratified by age (AOSpine international cohorts)	Surgical decompression	Not applicable (prognostic observational study)	Peri-operative → 2-year follow-up	mJOA recovery; complications by age	Elderly patients achieved neurological improvement but lower recovery ratios and higher complication rates than younger patients, supporting age-tailored peri-operative surveillance	JBI (Cohort)	11/11 (High)
Nagoshi et al., (2017)	Multicentre	Prospective cohort	Prognostic	470	Adults undergoing anterior cervical surgery for DCM (multicentre)	Anterior cervical decompression/fusion	Not applicable (risk-factor observational study)	Early post-op	Dysphagia incidence and predictors	Identified incidence and predictors of post-operative dysphagia after anterior surgery, informing nursing swallowing assessment and aspiration precautions	JBI (Cohort)	11/11 (High)
Fehlings et al. (2017)	International	Clinical practice guideline	Guideline / consensus	–	Adults with mild/moderate/severe DCM and non-myelopathic cord compression	Surgical decompression (guideline scope)	Clinical practice guideline recommendations (management pathway)	Whole peri-operative pathway	Graded clinical recommendations	Recommends surgery for moderate–severe DCM and offers structured management guidance, anchoring evidence-based peri-operative and rehabilitation planning	AGREE II	High (D1–D6: 95/88/92/96/85/92%)
Ren et al., (2017)	China	Retrospective cohort	Prognostic	132	Adults with CSM (ACDF vs laminoplasty comparison)	ACDF vs laminoplasty	Not applicable (comparative observational study)	Post-op recovery trajectory	Neurological recovery pattern	Compared recovery trajectories between anterior and posterior approaches, contextualising expected functional course during nursing rehabilitation	JBI (Cohort)	9/11 (Moderate)
McLynn et al., (2018)^b^	USA	Retrospective cohort	Prognostic	109,609	Adults undergoing elective spine surgery (large registry)	Elective spine surgery	Not applicable (risk-factor observational study)	Peri-operative	VTE incidence and predictors	Quantified VTE incidence and risk factors in elective spine surgery, supporting risk-stratified thromboprophylaxis and surveillance	JBI (Cohort)	11/11 (High)
Nagoshi et al., (2019)	Japan	Retrospective cohort	Prognostic	126	Adults after cervical laminoplasty for DCM	Cervical laminoplasty	Not applicable (observational study, JOACMEQ)	Post-op	JOACMEQ-based clinical indicators; surgical outcomes	Defined clinical indicators of outcome after laminoplasty using the JOACMEQ, supporting functional monitoring instruments in follow-up	JBI (Cohort)	11/11 (High)
Zakaria et al., (2019)	USA	Retrospective cohort	Prognostic	8,236	Adults undergoing cervical spine surgery (MSSIC registry)	Cervical spine surgery	Not applicable (registry observational study)	90 days post-op	Complication/adverse-event rates and risk factors	Characterised 90-day adverse events and their predictors, informing acute-phase complication surveillance and discharge planning	JBI (Cohort)	11/11 (High)
Badhiwala et al., (2020)	International	Narrative review	Contextual	–	Adults with DCM (evidence synthesis)	Not applicable (narrative review)	Not applicable (disease update)	Whole disease course (contextual)	Disease understanding; future directions	Synthesised contemporary DCM understanding and research gaps, providing conceptual context for rehabilitation need	SANRA	10/12 (High)
Choi and Kang, (2020)	Korea	Narrative review	Contextual	–	Adults with DCM (evidence synthesis)	Not applicable (narrative review)	Not applicable (pathophysiology/treatment overview)	Whole disease course (contextual)	Treatment approaches	Reviewed DCM pathophysiology and treatment strategies, providing background for surgical-plus-rehabilitation care model	SANRA	8/12 (Moderate)
Bui et al., (2021)	USA	Retrospective cohort	Prognostic	35,832	Adults undergoing cervical spine surgery (large cohort)	Cervical spine surgery	Not applicable (risk-factor observational study)	Peri-operative	VTE incidence and risk factors	Reported VTE incidence and risk factors specific to cervical spine surgery, refining thromboprophylaxis targeting	JBI (Cohort)	9/11 (Moderate)
Davies et al., (2022)	International	Consensus	Guideline / consensus	429	International DCM stakeholders (n=429; RECODE-DCM consensus)	Not applicable (consensus)	Consensus-defined research priorities (incl. rehabilitation)	Whole pathway (research agenda)	Prioritised research questions	Established consensus research priorities, naming peri-operative rehabilitation as a key gap, directly motivating nurse-led rehabilitation research	AGREE II	High (D1–D6: 88/80/85/90/75/88%)
Nouri et al., (2022)	International	Narrative review	Prognostic	–	Adults with DCM (evidence synthesis)	Not applicable (narrative review)	Not applicable (natural-history/risk overview)	Whole disease course (contextual)	Risk stratification for myelopathy development	Reviewed natural history and risk factors for myelopathic progression, supporting preoperative risk stratification	SANRA	10/12 (High)
**Domain 2: Phased functional exercise rehabilitation (n = 14)**												
bb	Spain/USA	Systematic review	Secondary	5 studies (191)	Adults with spinal cord injury (5 studies, 191 participants)	Not applicable (systematic review)	Physical-therapy interventions targeting participation-level outcomes	Post-acute rehabilitation	Participation and functional outcomes	Synthesised physical-therapy effects on participation after SCI, supporting activity- and participation-focused rehabilitation goals transferable to myelopathy	AMSTAR 2	High
Kopjar et al., (2015)	USA/Canada	Validation	Measurement	277	Adults with DCM (validation cohort)	Surgical decompression cohort (instrument validation)	Not applicable (psychometric validation of mJOA)	Baseline/follow-up assessment	mJOA reliability, validity, responsiveness	Confirmed the mJOA as a valid, reliable outcome measure, underpinning standardised neurological monitoring in nursing follow-up	COSMIN	Adequate (High)
Kato et al., (2015)	Japan	Multicentre validation	Measurement	92	Adults with DCM (multicentre)	Surgical cohort (instrument comparison)	Not applicable (JOA vs mJOA comparison)	Baseline/follow-up assessment	Concordance of JOA and mJOA	Demonstrated comparability of JOA and mJOA scores, guiding consistent functional scoring across settings	COSMIN	Adequate (High)
Tetreault et al. (2015)	Multicentre	Prospective cohort	Prognostic	757	Adults with DCM (multicentre prospective)	Surgical decompression	Not applicable (prognostic/MCID study)	Baseline → 1–2 y follow-up	mJOA minimal clinically important difference	Established the mJOA MCID, enabling interpretation of clinically meaningful recovery, informing rehabilitation goal-setting	JBI (Cohort)	11/11 (High)
Tetreault et al. (2017)	Multicentre	Validation	Measurement	757	Adults with DCM (multicentre)	Surgical decompression	Not applicable (validation study)	Baseline assessment	mJOA severity thresholds (mild/moderate/severe)	Defined mJOA severity categories, supporting risk stratification and nursing-system selection	COSMIN	Adequate (High)
Badran et al. (2018)	UK	Systematic review	Secondary	1 study	Adults after DCM surgery (systematic review; 1 eligible study)	Post-DCM-surgery rehabilitation	Post-operative physiotherapy (as reviewed)	Post-operative rehabilitation	Recovery/functional evidence	Found only one eligible study and insufficient evidence to guide post-operative physiotherapy, defining the evidence gap this review addresses	AMSTAR 2	High
Kalsi-Ryan et al., (2020)	Canada	Validation	Measurement	169	Adults with DCM (instrument-development cohort)	Surgical cohort (assessment development)	Not applicable (GRASSP-M development)	Baseline/follow-up assessment	Upper-limb (hand) sensorimotor assessment sensitivity	Developed the GRASSP-M for sensitive hand-function assessment, supporting granular monitoring of self-care–relevant motor recovery	COSMIN	Adequate (High)
Cheng et al. (2020)	Taiwan	Quasi-experimental	Direct rehabilitation	29	Post-operative adults with DCM (n=29)	Prior surgical decompression	Perturbation-based balance training programme (4 weeks)	Stabilization phase (post-op)	Postural stability, gait speed, functional performance	Balance training improved postural stability (d=0.84), gait speed and function after 4 weeks, supporting targeted balance rehabilitation in the sub-acute phase	JBI (Quasi-exp.)	7/9 (Moderate)
Jannelli et al., (2020)	Switzerland	Narrative review	Contextual	–	Adults with DCM (evidence synthesis)	Surgical decompression (context)	Not applicable (review of predictors/multimodal care)	Peri-operative (contextual)	Prognostic factors	Reviewed outcome predictors and a multimodal management rationale, supporting individualised rehabilitation planning	SANRA	8/12 (Moderate)
Coronado et al. (2020)	USA	Pilot RCT	Direct rehabilitation	30	Adults after ACDF (pilot RCT, n=30)	Anterior cervical discectomy and fusion	Early self-directed home exercise programme with telephone support	Early post-op → stabilization phase	Feasibility, safety, pain, opioid use	Early telephone-supported home exercise was safe and feasible after ACDF, providing a practical hospital-to-home transition model	JBI (RCT)	9/13 (Moderate)
Boerger et al. (2022)	International	Narrative review	Contextual	–	Adults with DCM (RECODE-DCM narrative review)	Surgical decompression (context)	Not applicable (peri-operative rehabilitation research priority)	Whole peri-operative pathway	Research priorities	Identified peri-operative rehabilitation as an unexplored priority, directly motivating nurse-led intervention trials	SANRA	10/12 (High)
Lorentz et al. (2023)	USA	Retrospective cohort	Direct rehabilitation	220	Adults after cervical spine surgery (n=220)	Cervical spine surgery	Post-operative physical therapy (referral vs none)	Post-operative	Patient-reported outcomes	Post-operative physical therapy was associated with improved patient-reported outcomes, supporting structured referral to rehabilitation	JBI (Cohort)	9/11 (Moderate)
Uehara et al. (2022)	Japan	RCT	Direct rehabilitation	61	Adults with CSM or OPLL undergoing muscle-preserving laminoplasty (n=61; 33 exercise, 28 control)	Muscle-preserving cervical laminoplasty (C2–C7)	Early neck-extension isometric strengthening and cervical range-of-motion exercises for 3 months from postoperative day 2 (vs no exercise); no cervical collar	Early post-op → 3 months	Axial pain (VAS); cervical range of motion; muscle strength	Early cervical strengthening and range-of-motion exercise did not significantly reduce axial pain at 2 weeks or 3 months versus control, although it was safe and feasible, tempering expectations for exercise as an axial-pain preventive	JBI (RCT)	10/13 (High)
Rahman et al. (2024)	USA	Retrospective cohort	Direct rehabilitation	66	Adults undergoing surgery for DCM (n=66; 27 anterior, 36 posterior approach)	Cervical decompression ± fusion (anterior or posterior)	Post-surgical rehabilitation (cervical range-of-motion, stabilization, gait training, upper-limb strengthening, occupational therapy); timing of initiation examined	Post-op → 12 months	mJOA; NDI; SF-36 PCS at 12 months	Post-surgical therapy was independently associated with improved mJOA and SF-36 PCS at 12 months, and early therapy (within ∼6 weeks) was associated with greater functional gains, supporting timely, structured rehabilitation	JBI (Cohort)	9/11 (Moderate)
**Domain 3: Pain management and psychobehavioral support (n = 12)**												
bb	Denmark	Prospective cohort	Nursing	87	Adults after spine surgery (n=87)	Spine surgery	Not applicable (observational pain-management audit)	Early post-op	Pain prevalence; management gaps	Documented insufficient post-operative pain control and management gaps, supporting systematic nurse-led pain assessment	JBI (Cohort)	9/11 (Moderate)
Oh et al., (2017)	USA	Cross-sectional	Contextual	285	Adults with CSM (n=285)	Surgical/CSM cohort	Not applicable (QoL benchmarking, SF-36)	Cross-sectional	SF-36 quality of life	Benchmarked CSM quality-of-life burden against other chronic diseases, framing holistic rehabilitation targets	JBI (Cross-sectional)	7/8 (Moderate)
bb	USA	Prospective cohort	Prognostic	141	Adults after lumbar and cervical degenerative spine surgery (n=141)	Lumbar/cervical degenerative spine surgery	Not applicable (prognostic study; fear-of-movement)	Early post-op (6 wk) → 6 mo	Tampa Scale for Kinesiophobia; pain, disability, physical health	Early post-operative fear of movement predicted pain, disability and poorer physical health at 6 months, identifying a modifiable psychological target	JBI (Cohort)	11/11 (High)
bb	Switzerland	Prospective cohort	Prognostic	234	Adults after lumbar spinal stenosis surgery (n=234; LSOS)	Lumbar spinal stenosis surgery	Not applicable (prognostic study; fear-avoidance beliefs)	Baseline, 6 mo, 12 mo	Fear-avoidance beliefs; pain, disability	Persistent (not baseline) fear-avoidance beliefs predicted worse 12-month pain (OR 0.34), highlighting a critical window for psychological intervention	JBI (Cohort)	11/11 (High)
Tetreault et al., (2019)	Multicentre	Prospective cohort	Measurement	395	Adults with DCM (multicentre, n=395)	Surgical decompression	Not applicable (outcome-interpretation study)	Post-op follow-up	Objective functional vs self-reported outcomes	Revealed discrepancy between objective functional and self-reported outcomes, cautioning on outcome interpretation in rehabilitation	JBI (Cohort)	11/11 (High)
Passias et al., (2021)	USA	Retrospective cohort	Prognostic	123	Adults with CSM (n=123)	Surgical decompression	Not applicable (classification study)	Post-op	Radiographic–functional–neurological relationships	Related radiographic and functional-neurological status, informing functional classification for care planning	JBI (Cohort)	9/11 (Moderate)
Haddas et al., (2021a)	USA	Prospective cohort	Prognostic	90	Adults with CSM (n=90)	Surgical decompression	Not applicable (fear-avoidance and gait biomechanics)	Pre/post-op (to 12 mo)	Tampa Scale for Kinesiophobia; gait parameters	Linked fear-avoidance to altered gait biomechanics in CSM, supporting addressing movement fear to optimise gait recovery	JBI (Cohort)	9/11 (Moderate)
Haddas et al. (2021b)^b^	USA	Prospective cohort	Prognostic	405	Adults with degenerative spine pathology (n=405)	Degenerative spine (surgical candidates)	Not applicable (classification study; TSK + Demoralization Scale)	Pre-operative assessment	Tampa Scale for Kinesiophobia; Demoralization Scale; gait/balance	Derived kinesiophobia/demoralization thresholds identifying patients whose gait and balance dysfunction is amplified by psychological factors	JBI (Cohort)	9/11 (Moderate)
Sherrod et al., (2023)^a^	USA	Retrospective cohort	Prognostic	1,141	Adults after cervical spine surgery (n=1,141)	Cervical spine surgery	Not applicable (prognostic study)	12–24 months post-op	New neck pain incidence and predictors	Identified predictors of new post-operative neck pain at 12–24 months, supporting longer-term pain surveillance	JBI (Cohort)	11/11 (High)
Zhong et al. (2023)	China	Cross-sectional	Prognostic	85	Post-operative adults with CSM (n=85; Fujian, China)	Surgical decompression	Not applicable (cross-sectional survey; TSK, Zung SAS, VAS)	Post-op	Tampa Scale for Kinesiophobia (≥37); anxiety; pain	81.17% exhibited kinesiophobia (TSK≥37), exceeding lumbar-disc-herniation cohorts; identified associated factors, supporting targeted screening	JBI (Cross-sectional)	7/8 (Moderate)
Chen et al. (2024)	China	Cross-sectional	Prognostic	122	Post-operative adults with CSM (n=122; Fujian, China)	Surgical decompression	Not applicable (cross-sectional; TSK grouping)	≤24 h post-op → 3 mo	Tampa Scale for Kinesiophobia; JOA; NDI; anxiety (SAS); ADL; grip strength	Reported 75.4% kinesiophobia prevalence, negatively correlated with functional (JOA) recovery, single-centre estimate requiring multicentre confirmation	JBI (Cross-sectional)	7/8 (Moderate)
aa	USA (multicentre)	Retrospective cohort	Prognostic	1,141	Adults undergoing CSM surgery (n=1,141; multicentre QOD)	Surgical decompression	Not applicable (prognostic registry study)	Baseline → 3 and 12 mo	Depression/anxiety; patient-reported outcomes; MCID	∼29% had preoperative depression or anxiety, independently associated with worse neck-pain outcomes at 3 and 12 months, supporting preoperative psychological screening	JBI (Cohort)	11/11 (High)
**Domain 4: Long-term follow-up and telerehabilitation (n = 10)**												
Gibson et al., (2018)	USA	Narrative review	Contextual	–	Adults with DCM (evidence synthesis)	Not applicable (narrative review)	Not applicable (clinical review)	Whole disease course (contextual)	Disease overview	Provided a clinical overview of DCM, providing background for long-term monitoring and follow-up planning	SANRA	8/12 (Moderate)
McCormick et al., (2020)	USA	Narrative review	Contextual	–	Adults with CSM (evidence synthesis)	Not applicable (narrative review)	Not applicable (diagnosis/management guide)	Whole disease course (contextual)	Clinical guidance	Summarised CSM diagnosis and management, supporting structured surveillance and patient education	SANRA	8/12 (Moderate)
Dijkman et al. (2022)	Netherlands	Prospective cohort	Prognostic	42	Adults after DCM surgery (n=42; long-term)	Surgical decompression	Not applicable (long-term outcome study)	Up to 10 years post-op	mJOA long-term outcomes	Reported durable neurological outcomes at 10 years, with some late deterioration, supporting long-term monitoring for late decline	JBI (Cohort)	11/11 (High)
Hejrati et al., (2022)	Canada	Narrative review	Contextual	–	Adults with DCM (evidence synthesis)	Surgical/holistic care (context)	Not applicable (personalised-care review)	Whole pathway (contextual)	Individualised care principles	Argued for personalised, patient-centred DCM management, aligns with individualised nursing-system selection	SANRA	8/12 (Moderate)
Catz et al. (2024)	Israel	Retrospective cohort	Direct rehabilitation	116	Adults with DCM (n=116)	Surgical decompression	Comprehensive inpatient multidisciplinary rehabilitation	Post-op rehabilitation	Daily performance (SCI-ARMI); functional outcomes	Comprehensive rehabilitation improved daily performance (SCI-ARMI 57→78; p<0.001), with gains observed beyond those expected from neurological recovery alone, supporting structured multidisciplinary rehabilitation	JBI (Cohort)	9/11 (Moderate)
Huang et al., (2024)	USA	Narrative review	Contextual	–	Adults with cervical degenerative disease (evidence synthesis)	Endoscopic cervical spine surgery (context)	Not applicable (surgical-technique review)	Peri-operative (contextual)	Surgical technique evolution	Reviewed evolution of endoscopic cervical spine surgery, providing context for procedure-specific recovery expectations	SANRA	8/12 (Moderate)
Lee et al., (2024)	Korea	Narrative review	Contextual	–	Adults with DCM (evidence synthesis)	Surgical decision-making (context)	Not applicable (algorithmic-roadmap review)	Peri-operative (contextual)	Surgical decision pathway	Proposed an algorithmic roadmap for DCM surgical management, framing the surgical context preceding rehabilitation	SANRA	8/12 (Moderate)
bb	Spain	Meta-analysis	Secondary	8 studies	Adults with chronic neck pain (meta-analysis; 8 studies)	Not applicable (meta-analysis)	Telerehabilitation programmes	Community/telehealth	Pain; disability	Telerehabilitation reduced pain and disability in chronic neck pain, supporting remote nurse-coordinated rehabilitation delivery	AMSTAR 2	High
Zhou et al., (2023)	China	Meta-analysis	Secondary	17 studies	Adults with multilevel CSM (meta-analysis; 17 studies)	Anterior vs posterior surgery	Not applicable (comparative meta-analysis)	Peri-operative → follow-up	Treatment outcomes by approach	Compared anterior vs posterior approaches for multilevel CSM, informing procedure-specific recovery and rehabilitation planning	AMSTAR 2	High
Wang et al., (2023)	China	Retrospective cohort	Nursing	257	Elderly adults undergoing cervical ACDF (n=257)	Anterior cervical discectomy and fusion	Enhanced Recovery After Surgery (ERAS) nursing pathway (multimodal peri-operative nursing: education, early mobilisation, pain/nutrition management)	Peri-operative → early recovery	ERAS outcomes (recovery, complications, length of stay)	ERAS nursing improved peri-operative recovery outcomes in elderly ACDF patients, demonstrating nurse-led pathway benefits	JBI (Cohort)	9/11 (Moderate)

a Sherrod et al. (2023) and Chan et al. (2023) draw on the same Quality Outcomes Database cervical spondylotic myelopathy cohort (n = 1,141) but examine different outcomes (new postoperative neck pain and the influence of depression/anxiety, respectively); they are reported as two analyses of one registry, not as independent cohorts.

b Adjacent-population (Tier 2) evidence. These studies were conducted in mixed or non-cervical spinal surgical cohorts, in spinal cord injury, or in chronic neck pain, and were included as transferable evidence under the three conditions set out in the eligibility criteria. They were appraised with the same design-appropriate instruments as the remaining studies, and no practice-oriented statement in the Results rests on them alone; statements drawing on them are identified as transferable in the Results. Of the 51 included studies, 42 (82.4%) are Tier 1 (core) evidence and 9 (17.6%) are Tier 2. **Note:** Studies are grouped by synthesis domain. Evidence type indicates each study’s role in the synthesis: direct rehabilitation evidence (delivering a rehabilitation intervention), nursing evidence (nurse-led care processes or protocols), prognostic evidence (predictors or outcomes), measurement evidence (instrument development or validation), guideline or consensus evidence, secondary evidence (systematic reviews and meta-analyses), and contextual evidence (narrative reviews and background studies). Direct rehabilitation and nursing studies provide the primary evidence for the practice-oriented findings; the remaining types support risk stratification, outcome interpretation and the Orem-based framework rather than direct intervention recommendations. In the n column, “–” denotes designs for which a participant count does not apply (e.g., reviews, guidelines) or was not reported; “Not applicable” in the Intervention column marks studies without a discrete rehabilitation/nursing intervention, whose contribution appears under Population, Outcome measures and Key findings. Methodological quality was appraised with the design-appropriate tool: JBI checklists (trials, cohort, cross-sectional, quasi-experimental), AMSTAR 2 (systematic reviews and meta-analyses), AGREE II (guidelines and consensus statements), COSMIN (measurement/validation), and SANRA (narrative reviews); the tool and score for each study appear in the final two columns and correspond to the item-level judgments in Supplementary Material S2. **Abbreviations:** ACDF, anterior cervical discectomy and fusion; ADL, activities of daily living; AGREE II, Appraisal of Guidelines for Research and Evaluation II; AMSTAR 2, A MeaSurement Tool to Assess systematic Reviews 2; AOSpine, AO Spine; COSMIN, COnsensus-based Standards for the selection of health Measurement INstruments; CSM, cervical spondylotic myelopathy; d, Cohen’s d; DCM, degenerative cervical myelopathy; DVT, deep vein thrombosis; ERAS, Enhanced Recovery After Surgery; GRASSP-M, Graded Redefined Assessment of Strength, Sensibility and Prehension – Myelopathy version; JBI, Joanna Briggs Institute; JOA, Japanese Orthopaedic Association; JOACMEQ, Japanese Orthopaedic Association Cervical Myelopathy Evaluation Questionnaire; LSOS, Lumbar Stenosis Outcome Study; MCID, minimal clinically important difference; mJOA, modified Japanese Orthopaedic Association; MSSIC, Michigan Spine Surgery Improvement Collaborative; NDI, Neck Disability Index; NRS, Numeric Rating Scale; OPLL, ossification of the posterior longitudinal ligament; OR, odds ratio; PCS, physical component summary; PE, pulmonary embolism; QOD, Quality Outcomes Database; RCT, randomized controlled trial; RECODE-DCM, REsearch objectives and Common Data Elements for DCM; ROM, range of motion; SANRA, Scale for the Assessment of Narrative Review Articles; SAS, Self-rating Anxiety Scale; SCI, spinal cord injury; SCI-ARMI, Spinal Cord Injury Ability Realization Measurement Index; SF-36, 36-item Short-Form Health Survey; TSK, Tampa Scale for Kinesiophobia; VAS, visual analogue scale; VTE, venous thromboembolism.

#### 3.4.2 Exclusion criteria

The exclusion criteria are those registered and were applied unchanged. Studies were excluded if they: (1) focused exclusively on the non-surgical management of cervical spondylotic myelopathy or degenerative cervical myelopathy; (2) enrolled patients with traumatic or neoplastic myelopathy; (3) constituted conference abstracts or case reports with fewer than five patients; or (4) were published in languages other than English or Chinese.

### 3.5 Study selection

The study selection process adhered to PRISMA guidelines (Fig. 2). The database search identified 5,235 records across five databases: PubMed/MEDLINE (n = 454), Embase (n = 779), CINAHL Complete (n = 208), Web of Science Core Collection (n = 833), and OpenAlex (n = 2,961). Following removal of 1,040 duplicate records using a custom deduplication script implemented in Python (pandas): records were matched first on Digital Object Identifier (DOI) and then, for records lacking a DOI, on a normalised title key (lower-casing, removal of punctuation and extra whitespace, and Unicode normalisation); all automated duplicate decisions were manually verified by one reviewer, 4,195 unique records underwent title and abstract screening by two independent reviewers. Initial disagreements occurred in approximately 8% of title/abstract decisions and 6% of full-text decisions; all discrepancies were resolved through discussion until consensus was achieved. This screening excluded 4,008 records. The full text of 187 reports was sought; 10 could not be retrieved, leaving 177 reports assessed for eligibility. Of these, 126 were excluded for the following reasons: not rehabilitation-focused (n = 58), ineligible population (n = 31), outcomes not relevant (n = 23), duplicate report of an included study (n = 11), non-surgical management only (n = 2), and trial protocol without outcome data (n = 1). The last of these was the published protocol of a randomized controlled trial of virtual-reality–augmented rehabilitation after surgery for cervical spondylotic myelopathy (Wu et al., 2024); it was excluded because it reports no outcome data and a trial protocol is not among the eligible study designs, and no publication reporting the results of the trial was identified. Ultimately, 51 studies met all inclusion criteria.

### 3.6 Data extraction

Data were extracted independently by two reviewers using a standardized form developed a priori and pilot-tested on five articles. Extracted information encompassed: study characteristics (author, year, country, design, sample size); population characteristics (age, sex, diagnosis, surgical procedure); intervention details following the Template for Intervention Description and Replication checklist where applicable (Hoffmann et al., 2014); outcome measures; and key findings pertinent to rehabilitation nursing practice. Discrepancies were resolved through consensus discussion.

### 3.7 Quality appraisal

Methodological quality was evaluated using Joanna Briggs Institute (JBI) Critical Appraisal Tools appropriate for each study design (Aromataris, 2020). Systematic reviews were appraised using A MeaSurement Tool to Assess systematic Reviews 2 (AMSTAR 2; Shea et al., 2017), and clinical guidelines using the Appraisal of Guidelines for Research and Evaluation II (AGREE II) instrument (Brouwers et al., 2010). Validation studies were appraised using the COnsensus-based Standards for the selection of health Measurement INstruments (COSMIN) risk-of-bias checklist, and narrative reviews using the Scale for the Assessment of Narrative Review Articles (SANRA). Two reviewers independently assessed study quality, with discrepancies resolved through discussion. Quality appraisal served to contextualize evidence strength in the synthesis. Tier 1 and Tier 2 studies were appraised with the same design-appropriate instruments and are reported together. Detailed quality appraisal results are provided in Supplementary Material S2.

### 3.8 Data synthesis

Given the heterogeneity in study designs, interventions, and outcomes, a narrative synthesis was conducted following the guidance of Popay et al. (2006). Thematic analysis was performed using the approach delineated by Braun and Clarke (2023). Two reviewers independently read and coded the extracted data. Initial codes were aggregated into preliminary domains, refined through iterative discussion, and mapped onto Orem’s theoretical constructs. Orem's Self-Care Deficit Nursing Theory was applied as an a priori analytical framework. During coding, reviewers independently mapped the emerging domains to Orem's three nursing systems (wholly compensatory, partly compensatory, supportive-educative) based on the level of patient self-care agency described in each study. Discrepancies in theoretical mapping were resolved through discussion until consensus was achieved. This deductive-inductive approach allowed the framework to structure the synthesis while remaining responsive to patterns emerging from the data. The four synthesis domains were finalized upon achieving consensus between reviewers.

## 4 Results

A total of 51 studies met the inclusion criteria. Thematic analysis organised the evidence into four cross-cutting synthesis domains: (1) risk stratification and acute-phase management, (2) phased functional exercise rehabilitation, (3) pain management and psychobehavioural support, and (4) long-term follow-up and telerehabilitation. Each of these spans, rather than corresponds to, the postoperative course. That course is described as four temporal rehabilitation phases: protective (days 1–14), stabilization (weeks 2–6), integration (weeks 6–12), and long-term follow-up (>12 weeks). Orem's three nursing systems map onto this temporal progression, being wholly compensatory in the protective phase, partly compensatory during stabilization, and supportive-educative during integration and long-term follow-up, independently of domain. Table 1 presents the characteristics of the included studies by synthesis domain, and Fig. 3 shows the resulting two-dimensional structure (domains × phases). The four subsections below report the synthesis for each domain.

**Fig. 3:**
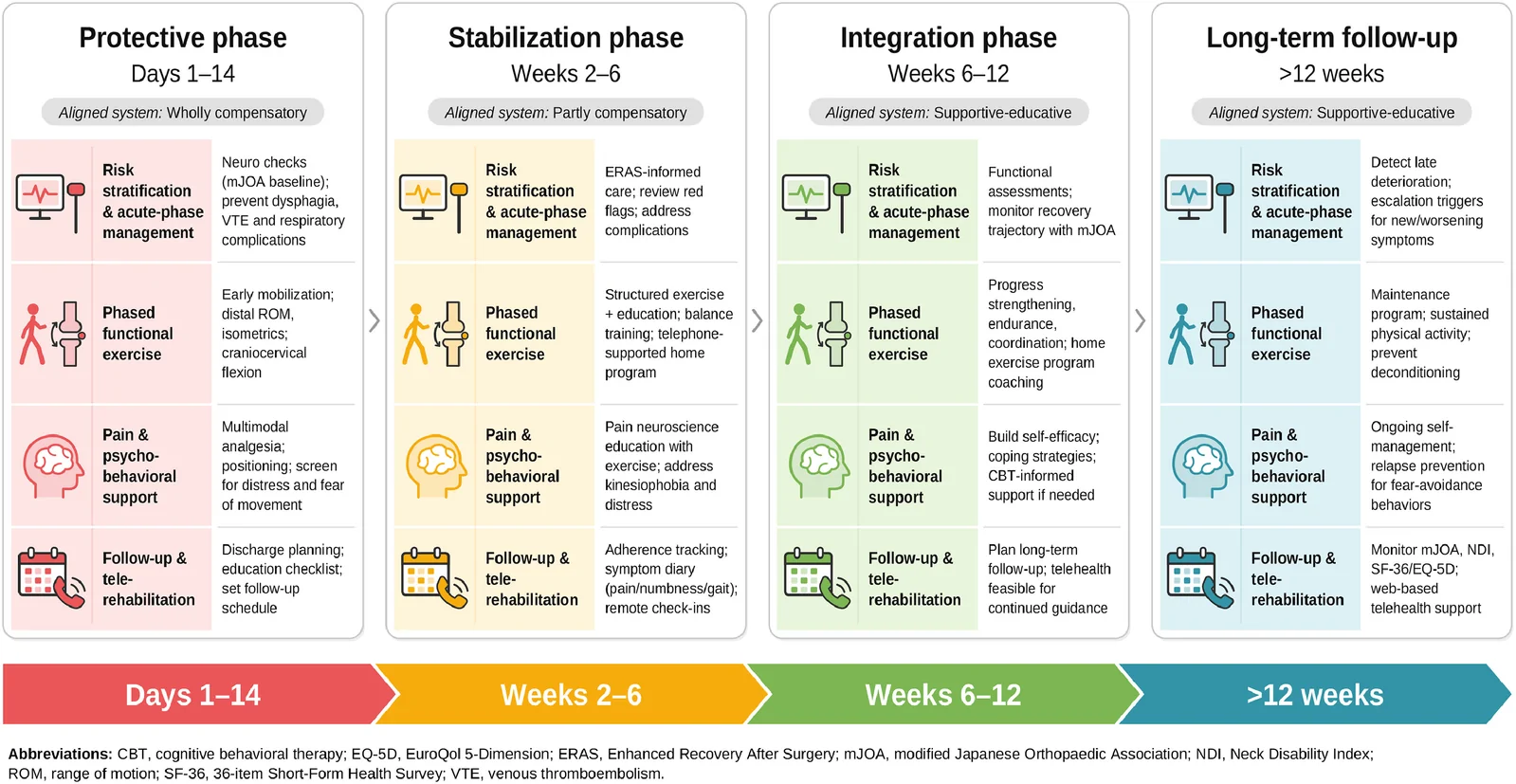
**Conceptual synthesis of postoperative rehabilitation nursing for cervical spondylotic myelopathy, informed by both the included studies and contextual literature.** The Fig. is arranged as a two-dimensional structure in which the four synthesis domains (risk stratification & acute-phase management; phased functional exercise; pain & psychobehavioural support; follow-up & telerehabilitation) are shown across four temporal rehabilitation phases (protective, days 1–14; stabilization, weeks 2–6; integration, weeks 6–12; long-term follow-up, >12 weeks). Each phase card lists representative nursing actions for every domain, and neutral tags indicate the Orem nursing system predominating in that phase (wholly compensatory, partly compensatory, supportive-educative). The bottom ribbon depicts the chronological progression. This Fig. was redrawn as vector artwork with the assistance of Claude (Anthropic); the authors reviewed and verified its content.

### 4.1 Domain 1: Risk stratification and acute-phase management

Domain 1 (risk stratification and acute-phase management) synthesises evidence on peri-operative risk and early complication surveillance. Although this evidence bears predominantly on the early postoperative course, that is, the protective phase (days 1–14), when nursing care is largely wholly compensatory as patients regain self-care capacity, the domain remains thematic rather than time-bound, and its risk-stratification logic also informs later phases.

#### 4.1.1 Preoperative risk factors

Among the included studies, Domain 1 addressed risk stratification and acute-phase surveillance (Table 1); preoperative characteristics that may shape recovery, including psychological distress, are examined as contextual evidence in the Discussion. Within the included corpus, patient age emerged as an important determinant of surgical outcome and perioperative risk, and validated instruments were established for grading impairment and tracking recovery (Table 1). Guidelines and prognostic evidence support surgical decompression for moderate-to-severe disease and define modified Japanese Orthopaedic Association severity thresholds that inform risk stratification (Fehlings et al., 2017; Tetreault et al., 2015, 2017). These included study findings that point to the need for age-tailored, risk-stratified peri-operative nursing surveillance. Broader contextual evidence on the magnitude of surgical benefit and on modifiable preoperative risk factors, such as symptom duration, sarcopenia, and psychological distress, is examined more fully in the Discussion.

#### 4.1.2 Postoperative surveillance and complication prevention

Systematic neurological assessment enables early identification of surgical complications; documentation using validated instruments, such as the modified Japanese Orthopaedic Association score and the Japanese Orthopaedic Association Cervical Myelopathy Evaluation Questionnaire, establishes baseline function and tracks the recovery trajectory (Tetreault et al., 2017). Across the included cohorts, the acute phase carried a measurable complication burden: registry and prospective studies characterised 90-day adverse events and showed that older patients experienced more complications, together defining priorities for acute-phase nursing surveillance (Table 1).

Venous thromboembolism was the most frequently studied acute complication in the included corpus. Prospective and registry cohorts characterised its incidence and risk factors and evaluated prophylaxis, supporting risk-stratified mechanical and pharmacological thromboprophylaxis, together with early mobilisation, as core nursing responsibilities (Akeda et al., 2014; Table 1); much of this evidence derives from mixed spinal-surgical rather than myelopathy-specific cohorts and is therefore transferable rather than directly applicable. Dysphagia after anterior cervical surgery was a further nursing priority: an included multicentre cohort identified its incidence and predictors, informing structured swallowing assessment and aspiration precautions (Table 1). Contextual evidence on the natural course of post-operative dysphagia, on respiratory-complication prevention, and on other acute-phase nursing concerns (urinary management and pressure-injury prevention) is addressed in the Discussion. Within the proposed framework, this domain supplies the assessment inputs (age, myelopathy severity grade, and complication risk) that determine the initial nursing system and the intensity of early surveillance.

### 4.2 Domain 2: Phased functional exercise

Functional exercise rehabilitation follows a phased approach, with nursing systems transitioning from wholly compensatory toward supportive-educative as patient self-care agency increases.

#### 4.2.1 Evidence base for rehabilitation

The included secondary evidence indicates that direct evidence for post-operative rehabilitation in cervical spondylotic myelopathy remains sparse. A systematic review found only one eligible trial and insufficient evidence to guide post-operative physiotherapy (Badran et al., 2018; Table 1), and a rehabilitation-focused synthesis in a related spinal-cord population supported activity- and participation-oriented goals transferable to myelopathy (Gómara-Toldrà et al., 2014; Table 1). Consistent with this gap, the AO Spine RECODE-degenerative cervical myelopathy consortium identified peri-operative rehabilitation as an under-researched priority (Boerger et al., 2022; Table 1). Among the included primary studies, an observational cohort found that post-surgical therapy was independently associated with improved function (modified Japanese Orthopaedic Association and 36-item Short-Form Health Survey physical component summary) at 12 months, with earlier initiation associated with greater gains (Rahman et al., 2024; Table 1). In sum, the included evidence supports the plausibility and priority of structured post-operative rehabilitation, while indicating that high-quality primary trials remain scarce.

#### 4.2.2 Protective phase (days 1–14)

The protective phase prioritises neural-tissue healing while limiting deconditioning, with nursing care predominantly wholly compensatory. Direct included evidence for this phase is limited to a single randomized controlled trial: early cervical isometric strengthening and range-of-motion exercise begun on the second post-operative day was safe and feasible but did not reduce axial pain at 2 weeks or 3 months compared with usual care (Uehara et al., 2022; Table 1). This null result cautions against assuming that early exercise prevents axial pain, and indicates that, in this phase, nursing emphasis appropriately remains on protected mobilisation and complication prevention rather than on pain-preventive exercise.

#### 4.2.3 Stabilization phase (weeks 2–6)

As self-care agency returns, nursing care shifts from wholly to partly compensatory, and the included intervention studies converge on the feasibility and functional value of structured exercise in this window. An early telephone-supported home exercise programme after anterior cervical discectomy and fusion was safe and feasible and supported the hospital-to-home transition (Coronado et al., 2020; Table 1). Perturbation-based balance training improved postural stability (d = 0.84), gait speed and functional performance over four weeks in a post-operative myelopathy cohort (Cheng et al., 2020; Table 1). Comprehensive multidisciplinary rehabilitation was associated with improved daily performance (Spinal Cord Injury Ability Realization Measurement Index 57→78; p < 0.001), with gains observed beyond those expected from neurological recovery alone (Catz et al., 2024; Table 1). These included studies indicate that targeted, multimodal exercise in the sub-acute phase is feasible and associated with functional improvement, though the evidence comprises small trials and observational cohorts.

#### 4.2.4 Integration phase (weeks 6–12)

The integration phase aligns with a progressively supportive-educative nursing role centred on self-management education, home-exercise instruction and follow-up planning. Included evidence specific to this phase is limited: the home-based transitional model of Coronado et al. (2020; Table 1) is most applicable, and post-operative physical therapy was associated with improved patient-reported outcomes in a cervical-surgery cohort (Lorentz et al., 2023; Table 1). The paucity of included studies addressing this phase is a finding in its own right, and marks it as a priority for future nurse-led intervention research. This domain, in turn, provides the graded exercise and educational content through which the framework operationalises the shift from wholly to partly compensatory and, ultimately, supportive-educative care as self-care agency increases.

### 4.3 Domain 3: Pain management and psychobehavioral support

#### 4.3.1 Multimodal pain management

Within the included corpus, evidence on post-operative pain management specific to cervical spondylotic myelopathy is limited but points to a persistent care gap: an audit of post-operative pain documented insufficient pain control and management gaps, supporting systematic, nurse-led pain assessment as a core responsibility (Nielsen et al., 2014; Table 1). Broader evidence on multimodal analgesia and on interventions that improve pain self-efficacy in non-populations with cervical spondylotic myelopathy is drawn on as contextual evidence in the Discussion.

#### 4.3.2 Kinesiophobia and psychological distress

Two included single-centre cross-sectional studies from China report a high prevalence of kinesiophobia after surgery for cervical spondylotic myelopathy, at 75.4% (Chen et al., 2024; Table 1) and 81.17% (Tampa Scale for Kinesiophobia ≥ 37; Zhong et al., 2023; Table 1), both exceeding rates reported in lumbar-disc cohorts, although their single-centre design limits generalisability. Included prognostic evidence links fear of movement to worse recovery: early post-operative kinesiophobia predicted pain, disability and poorer physical health at 6 months (Archer et al., 2014; Table 1), and persistent, rather than baseline, fear-avoidance beliefs predicted worse 12-month pain (Burgstaller et al., 2017; Table 1), identifying a critical window for psychological intervention; both cohorts were drawn from adjacent spinal-surgical populations, so this association is transferable rather than myelopathy-specific. In a myelopathy-specific cohort, fear-avoidance was linked to altered gait biomechanics, indicating that fear of movement has observable functional correlates (Haddas et al., 2021a; Table 1); complementary threshold work in an adjacent degenerative-spine cohort identified the levels of kinesiophobia and demoralization at which gait and balance dysfunction is amplified (Haddas et al., 2021b; Table 1). Conceptualised within Orem's framework, kinesiophobia represents a deficit in the motivational-volitional domain of self-care agency and a modifiable target for nursing assessment and support. The broader lumbar and general spine-surgery fear-avoidance literature, and the reported prevalence of preoperative psychological distress, are taken up as contextual evidence in the Discussion. For the framework, the contribution of this domain is to identify the motivational-volitional deficits (kinesiophobia and psychological distress) that nurses must assess and address if patients are to progress between nursing systems.

### 4.4 Domain 4: Long-term follow-up and telerehabilitation

Domain 4 (long-term follow-up and telerehabilitation) synthesises evidence on sustaining functional gains beyond discharge and translating hospital-based improvement into lasting independence. This evidence maps chiefly onto the long-term follow-up phase (>12 weeks), in which nursing is predominantly supportive-educative and patients assume primary responsibility for self-care with nursing guidance and support.

Included long-term evidence shows durable neurological outcomes at 10 years with some late deterioration, supporting sustained monitoring for late decline (Dijkman et al., 2022; Table 1), while comprehensive rehabilitation and post-operative physical therapy were associated with improved long-term and patient-reported outcomes (Lorentz et al., 2023; Catz et al., 2024; Table 1). For remote delivery, an included meta-analysis found that telerehabilitation reduced pain and disability in adults with chronic neck pain, supporting nurse-coordinated remote rehabilitation as a transferable rather than myelopathy-specific delivery model (Valenza-Peña et al., 2024; Table 1), and a nurse-led enhanced-recovery pathway improved peri-operative recovery in older adults undergoing anterior cervical discectomy and fusion, illustrating a feasible model of structured nursing support (Wang et al., 2023; Table 1). Nurses are well positioned to coordinate telerehabilitation, conducting remote assessments, guiding exercise, and monitoring patient-reported outcomes; broader telerehabilitation and outcome-measurement literature is considered contextually in the Discussion. This domain, finally, specifies the supportive-educative monitoring and telerehabilitation activities through which the framework sustains self-care and detects late deterioration beyond discharge.

## 5 Discussion

This integrative review synthesised current evidence on postoperative rehabilitation nursing for patients with cervical spondylotic myelopathy and, using Orem's Self-Care Deficit Theory, organised it into four synthesis domains: risk stratification and acute-phase management; phased functional exercise rehabilitation; pain management and psychobehavioural support; and long-term follow-up and telerehabilitation. Across these domains, the principal contribution of the review is an integrated, theory-informed account of the nursing role along the whole postoperative pathway, from acute-phase surveillance and complication prevention, through progressive functional exercise, to community-based follow-up and telerehabilitation. Within this account, psychological barriers such as kinesiophobia and preoperative psychological distress, reported by a limited number of predominantly single-centre studies, represent one relevant component that falls within nursing's scope of practice, rather than the central finding of the review.

Read together, the four domains describe a continuous, theory-guided nursing trajectory. In the acute phase, the included cohorts foreground risk-stratified surveillance and complication prevention (venous thromboembolism, dysphagia, and other early adverse events), consistent with Orem's wholly compensatory system. As patients regain capacity, phased functional-exercise evidence, including balance training and telephone-supported home-exercise programmes, supports a graded transition through partly compensatory to supportive-educative care. Pain and psychobehavioural evidence indicates that unrelieved pain and movement-related fear can impede engagement in rehabilitation, positioning multimodal pain management and psychological screening as enablers of self-care agency across phases. Finally, long-term follow-up and telerehabilitation evidence extends the nursing role beyond discharge, helping to sustain functional gains and self-management in the community. This cross-domain synthesis, rather than any single finding, constitutes the review's contribution.

The proposed Rehabilitation Nursing System Selection Framework provides a structured approach for applying Orem's theory to clinical practice. This framework offers nurses a systematic method for assessing patient self-care agency and selecting appropriate nursing interventions across the rehabilitation trajectory.

### 5.1 Multifactorial nature of recovery from cervical spondylotic myelopathy

Effective rehabilitation after surgery for cervical spondylotic myelopathy involves multiple factors that extend well beyond surgical intervention. Surgical advances have improved immediate outcomes: large prospective studies report significant neurological improvement (Fehlings et al., 2013), and meta-analyses support benefit even in elderly populations (Machino et al., 2012; Madhavan et al., 2016). The findings that sarcopenia affects two-year outcomes (Gong et al., 2025) and that prolonged symptom duration predicts inferior surgical outcomes (Karpova et al., 2013), including quality of life (Levett et al., 2026) reveal that preoperative patient characteristics shape long-term recovery trajectories. These risk factors are amenable to preoperative assessment and may be partially modifiable through targeted interventions, a consideration with direct implications for prehabilitation program design.

As contextual evidence beyond the included studies, several reports underscore the prognostic weight of preoperative characteristics. Sarcopenia has been associated with inferior pain relief and functional recovery at two-year follow-up (Gong et al., 2025); preoperative psychological distress, reported in approximately one-third (31%) of patients with cervical spondylotic myelopathy, has been linked to poorer surgical outcomes (Wang et al., 2025); and prolonged symptom duration has been associated with a ‘pain-dominant’ phenotype and distinct recovery trajectories despite neurological improvement (Pedro et al., 2024). These observations are presented as contextual evidence rather than as findings of the present synthesis, and they reinforce the value of preoperative, nurse-led assessment of modifiable physical and psychological risk factors.

As further contextual evidence beyond the included corpus, several reports inform acute-phase nursing priorities that fall outside the review's included studies. Older age has been associated with a higher risk of peri-operative complications (Veeravagu et al., 2016). Post-operative dysphagia after anterior cervical discectomy and fusion is common early but typically declines over the first post-operative year (Bazaz et al., 2002; Fountas et al., 2007), surgical-technique refinements such as prevertebral fascia suturing may reduce its incidence (Huang et al., 2025), and overall complication rates for this procedure have been reported in the region of 13–19% (Epstein, 2019). Prophylactic respiratory physiotherapy has been shown to reduce post-operative pulmonary complications in general peri-operative populations (Odor et al., 2020). Further recurring acute-phase nursing concerns include venous thromboembolism prophylaxis (Sebastian et al., 2016), urinary management with selective catheterisation in high-risk patients (Jung et al., 2013), and pressure-injury prevention, where hospital-acquired ulcers are associated with poorer motor recovery (Kopp et al., 2024) and telemedicine-supported and sensor-based approaches are emerging (Chen et al., 2022; Xiang et al., 2024). These observations are presented as contextual evidence rather than as findings of this synthesis, and they reinforce the value of proactive, nurse-led surveillance of airway, swallowing, respiratory, thromboembolic, urinary, and skin-integrity risks during the acute phase.

As contextual evidence beyond the included studies, several further bodies of literature inform the functional-exercise, pain, psychobehavioural and follow-up domains without forming part of the appraised corpus. For early post-operative mobilisation and cervical exercise, evidence from adjacent spinal and neck-pain populations describes graded range-of-motion, isometric and deep-cervical-flexor programmes (Blomgren et al., 2018; Barbosa et al., 2023), for which movement-control testing methods are also available (Bocos-Corredor et al., 2024). For pain management, the broader literature supports multimodal and regional analgesia (De Liyis et al., 2024; Yu et al., 2025), pain neuroscience education (Malfliet et al., 2018), and behavioural programmes that improve pain self-efficacy in chronic musculoskeletal pain (Martinez-Calderon et al., 2020; Rondon-Ramos et al., 2020; Davin et al., 2022). The wider spine-surgery literature indicates that fear-avoidance beliefs predict pain and disability and are amenable to cognitive-behavioural physiotherapy, predominantly in lumbar cohorts (Archer et al., 2011, 2016; Rolving et al., 2015; Fors et al., 2021), and that preoperative fear of movement is associated with later sedentary behaviour (Jakobsson et al., 2023); nurse-led and digital self-management approaches have improved psychological outcomes in adjacent neurological populations (Leo et al., 2022; Wu et al., 2025). For remote delivery and long-term monitoring, telerehabilitation has shown feasibility and effectiveness comparable to usual care (Cottrell et al., 2017; Brehon et al., 2022; Tan et al., 2025), and validated outcome instruments are available to support remote surveillance for late deterioration (Ware and Sherbourne, 1992; Brito et al., 2023). A recent secondary source on rehabilitation for degenerative cervical myelopathy (Smith et al., 2025), published after the search window, is consistent with these directions but was not eligible for inclusion. These sources are presented as contextual evidence and were not appraised as part of the 51 included studies.

### 5.2 Psychological factors in postoperative rehabilitation

Within the third of the four synthesis domains (pain management and psychobehavioural support), psychological barriers emerged as a recurring finding across the synthesised evidence, and are discussed here as one component of the broader nursing role rather than as the review's central finding. A limited number of, predominantly single-centre, studies reported high kinesiophobia (75.4%) and preoperative psychological distress (31%); while these specific estimates should be interpreted cautiously, they are consistent with a broader pattern of fear-avoidance and psychological burden reported across the spine-surgery literature. While these estimates derive primarily from single-center studies conducted in China (Chen et al., 2024; Wang et al., 2025), accumulating evidence from both myelopathy-specific investigations and the broader spine surgery literature provides triangulated support for the generalizability of this psychological burden.

Myelopathy-specific evidence corroborates these findings. Stoffman et al. (2005), in a seminal Canadian cohort study of 89 patients with cervical spondylotic myelopathy, documented that 29% exhibited depressive mood and 38% exhibited anxious mood, with psychological distress strongly associated with ambulatory dysfunction. More recently, Zhong et al. (2023) reported that 81.17% of postoperative patients with cervical spondylotic myelopathy in China exhibited kinesiophobia (Tampa Scale for Kinesiophobia ≥ 37), a prevalence exceeding that observed in lumbar disc herniation populations (67.13%). Chan et al. (2023), utilizing the Quality Outcomes Database, found that approximately 29% of patients undergoing surgery for cervical spondylotic myelopathy presented with either depression or anxiety preoperatively, with these psychological comorbidities independently associated with inferior neck pain outcomes at 3 and 12 months.

Converging evidence from degenerative spine surgery populations further substantiates these patterns. Svensson et al. (2011) reported a baseline prevalence, documenting elevated kinesiophobia in 45% of Swedish patients 10–34 months following lumbar disc herniation surgery. Archer et al. (2014) reported the prognostic significance of this phenomenon in a North American cohort of 141 patients undergoing lumbar and cervical degenerative spine surgery, finding that early postoperative kinesiophobia (at 6 weeks) significantly predicted pain intensity, disability, and diminished physical health at 6 months. Jakobsson et al. (2023) extended these findings longitudinally, revealing that preoperative fear of movement predicts sedentary behaviour at 12 months post-lumbar fusion (*β* = 0.013, 95% CI = 0.004–0.022, *p* = 0.007). Notably, Burgstaller et al. (2017) contributed a nuanced perspective from a Swiss multicenter cohort (n=234), showing that while preoperative fear-avoidance beliefs were not prognostic indicators, persistent fear-avoidance beliefs (present at both baseline and 6 months postoperatively) significantly predicted poorer pain outcomes at 12 months (OR 0.34, 95% CI: 0.16–0.73), underscoring the critical window for psychological intervention.

The convergence of evidence across myelopathy-specific and general spine surgery populations, spanning North American, European, and Asian healthcare contexts, suggests that the psychological burden identified in this review reflects a generalizable clinical phenomenon warranting systematic screening and targeted intervention strategies.

In interpreting these prevalence Fig.s, cross-cultural considerations are important. The highest kinesiophobia estimates in this review, at 75.4% (Chen et al., 2024) and 81.17% (Zhong et al., 2023), derive from Chinese single-centre cohorts and exceed Fig.s commonly reported in Western spine-surgery populations (e.g., 45%; Svensson et al., 2011). Several factors may contribute to this apparent difference. Culturally shaped health beliefs and illness behaviour, family-centred care expectations that can inadvertently reinforce activity avoidance, and variation in the timing and intensity of structured postoperative rehabilitation across health systems may all influence reported fear of movement. Measurement factors are also relevant: differences in translation and in the Tampa Scale for Kinesiophobia cut-off applied (≥ 37 in several Chinese studies) can affect prevalence estimates. These considerations suggest that the elevated Fig.s may partly reflect measurement and sampling rather than a purely cultural effect, and they reinforce the need for multi-centre, cross-cultural validation before prevalence estimates are generalised.

### 5.3 Evidence gaps and emerging directions

Our synthesis identified significant evidence gaps that warrant attention. Systematic reviews consistently note the paucity of rehabilitation research specific to degenerative cervical myelopathy (Badran et al., 2018; Smith et al., 2025), though emerging evidence from Rahman et al. (2024) suggests that rehabilitation timing influences outcomes. The identification of peri-operative rehabilitation as a research priority (Boerger et al., 2022) aligns with the need for nurse-led intervention trials. Telerehabilitation evidence (Cottrell et al., 2017; Brehon et al., 2022; Tan et al., 2025) supports extending nursing care beyond hospital confines, while digital self-management platforms (Leo et al., 2022) offer scalable intervention delivery mechanisms. Addressing these gaps requires a shift from fragmented interventions to integrated, theoretically grounded nursing care models.

### 5.4 Application of Orem's theory: A bidirectional dialogue between theory and evidence

This review applied Orem's Self-Care Deficit Theory as an a priori framework to guide evidence synthesis. The relationship between theory and evidence, however, proved bidirectional: while Orem's constructs structured the analysis, the synthesized evidence simultaneously illuminated how the theory requires adaptation for postoperative rehabilitation contexts.

The synthesized evidence showed notable alignment with Orem's theoretical propositions. Orem's tripartite conceptualization of nursing systems (wholly compensatory, partly compensatory, supportive-educative) corresponded well with the phased rehabilitation trajectory identified across studies: the immediate postoperative period aligned with wholly compensatory care, the stabilization phase with partly compensatory interventions, and long-term community reintegration with supportive-educative functions. This congruence suggests that Self-Care Deficit Nursing Theory provides a coherent organizational structure for postoperative rehabilitation nursing in this population, though prospective testing of this alignment in clinical practice remains warranted.

The synthesis also revealed dimensions requiring theoretical refinement, particularly regarding Orem's Power Components of Self-Care Agency. While Orem's original framework emphasises physical capability, we hypothesise, as an interpretive proposition derived from this synthesis rather than an established conclusion, that for a subset of patients with cervical spondylotic myelopathy the predominant barrier to early self-care may be cognitive-volitional as well as physical, rather than physical alone. This proposition requires prospective empirical testing. The elevated prevalence of kinesiophobia reported in recent studies, ranging from 75% to 81% in Chinese cohorts (Zhong et al., 2023; Chen et al., 2024), points to what may be understood within Orem's framework as a limitation in foundational capabilities and dispositions: a belief system in which movement is equated with harm. In theoretical terms, this represents a breakdown in the 'decision-making' and 'knowledge-seeking' power components. Patients may possess the physical capacity to move yet lack the agency to initiate movement due to fear.

This finding carries important implications for nursing system selection. Rather than defaulting to Wholly Compensatory care (protective interventions) based on observed functional limitations, nurses should consider early deployment of supportive-educative interventions, including therapeutic patient education and brief, psychologically-informed support that lies within nursing scope, to help restore patients' 'power to act'. More structured cognitive-behavioural techniques such as cognitive restructuring require specific competencies and are best delivered in collaboration with appropriately trained professionals, or by nurses with dedicated training, in keeping with interprofessional boundaries. This represents a potential refinement of Orem's framework for populations where psychological barriers predominate over physical deficits.

The proposed Rehabilitation Nursing System Selection Framework represents an attempt to bridge theory and practice: it retains Orem's core principles, namely systematic assessment of self-care agency, identification of deficits, and matched nursing system selection, while incorporating the psychological dimensions that this synthesis identified as particularly salient for populations with cervical spondylotic myelopathy. Future research should empirically test whether this adapted framework improves patient outcomes compared to standard care approaches.

### 5.5 Implications for nursing practice

Several concrete implications for nursing practice follow from this synthesis. First, psychological screening should be targeted and time-anchored. The Tampa Scale for Kinesiophobia (Woby et al., 2005; Dupuis et al., 2023) and the Hospital Anxiety and Depression Scale (Zigmond and Snaith, 1983) are appropriate for this population because myelopathic symptoms are frequently provoked by movement, which makes fear-avoidance particularly salient, and because anxiety and depressive symptoms are common around cervical spine surgery (Doi et al., 2019). In practice, nurses can administer a baseline Tampa Scale for Kinesiophobia and Hospital Anxiety and Depression Scale preoperatively, repeat them in the early postoperative period before first mobilisation, and again at each rehabilitation-phase transition and at follow-up. Commonly used thresholds can structure the nursing response: a Tampa Scale for Kinesiophobia-17 score at or above 37 flags clinically significant kinesiophobia that warrants graded activity, reassurance, and movement-confidence education rather than activity restriction; a Hospital Anxiety and Depression Scale subscale score of 8-10 indicates possible anxiety or depression warranting closer monitoring and low-intensity psychological support, while a score of 11 or above indicates a probable case that warrants referral. Persistently high scores, failure to progress functionally, or any expressed hopelessness or safety concern should prompt timely referral to psychology, psychiatry, or the pain service.

Second, assessment of self-care agency should be operationalised with observable indicators in each of the three domains and linked directly to the care plan. In the cognitive-knowledge domain, nurses can check whether the patient can state postoperative precautions, recognise warning signs (for example, worsening weakness, numbness, or gait disturbance), and describe the prescribed exercise plan. In the motivational-volitional domain, indicators include self-efficacy for exercise, adherence to the agreed plan, and the level of movement-related fear captured by the Tampa Scale for Kinesiophobia. In the physiological-functional domain, indicators include hand and grip function, gait and balance, and independence in activities of daily living. These are assessed on admission, daily during the acute phase, at each phase transition, and at follow-up. The domain that is weakest determines the nursing emphasis: knowledge gaps call for structured education and teach-back; volitional gaps call for motivational support, goal-setting, and graded exposure to feared movements; and physiological gaps call for assisted or guided practice and appropriate assistive strategies.

Third, transitions between Orem's nursing systems should follow explicit, reassessed criteria rather than elapsed time alone. A wholly compensatory approach is appropriate while the patient is dependent for mobility and activities of daily living or while neurological status is still unstable. Progression to a partly compensatory approach is indicated once the patient is haemodynamically and neurologically stable, able to participate in assisted exercise, and has adequately controlled pain. Progression to a supportive-educative approach is indicated once the patient can perform self-care and prescribed exercises with supervision, demonstrates adequate knowledge and self-efficacy on the indicators above, and is approaching discharge readiness. Documenting these criteria and reassessing them at defined intervals allows nurses to individualise the pace of progression and to step care back up if a patient deteriorates. Telerehabilitation can then extend supportive-educative care beyond discharge for patients who need continued guidance.

### 5.6 Implications for policy and interdisciplinary practice

This review also has implications for interdisciplinary collaboration and the organisation of care. Because the nursing role spans the whole postoperative pathway, it is important to specify who assesses what, and when. Nurse-led psychosocial and self-care-agency screening can be embedded at defined points (preoperatively, early postoperatively, at phase transitions, and at follow-up), with results shared in a common assessment record. From these assessments, nurses coordinate with the wider team along clear referral pathways: with surgeons on neurological change, complications, and the timing of mobilisation; with physiotherapists on the progression of functional exercise; with rehabilitation physicians on the overall rehabilitation plan; with psychologists or psychiatry when Tampa Scale for Kinesiophobia or Hospital Anxiety and Depression Scale thresholds are exceeded or when a patient fails to progress; and with community and telehealth services for follow-up and telerehabilitation. Defining the thresholds that trigger each referral, and the information passed on, helps make this coordination reliable rather than ad hoc, and multidisciplinary models of this kind have been associated with improved outcomes (Tamai et al., 2023).

At the policy level, these arrangements require enabling conditions. Care pathways can formalise the screening schedule, the nursing-system transition criteria, and the referral thresholds described above, so that they are applied consistently across patients and settings. This in turn calls for protected nursing time and brief training in the administration and interpretation of the Tampa Scale for Kinesiophobia and Hospital Anxiety and Depression Scale and in graded-exposure and motivational-support techniques, together with the infrastructure needed to deliver and reimburse telerehabilitation and nurse-led follow-up. Because the framework proposed here is conceptual, prospective evaluation of these nurse-led pathways, including their effect on functional and psychological outcomes and their cost-effectiveness, is a necessary next step before wide-scale adoption.

### 5.7 Strengths and limitations

A key strength of this review is the comprehensive search strategy spanning five major databases (including Embase and OpenAlex), which ensures comprehensive coverage of the global literature. However, several limitations warrant acknowledgment. First, the integrative methodology precludes meta-analysis, and conclusions derive from narrative synthesis rather than pooled effect estimates. Second, quality appraisal revealed moderate overall evidence strength, with limitations in randomized controlled trial sample sizes and blinding procedures. Third, key findings regarding kinesiophobia (75.4%) and psychological distress (31%) derive primarily from single-center studies conducted in China; although triangulation with Western spine surgery populations supports the generalizability of elevated kinesiophobia prevalence, direct cross-cultural validation in myelopathy-specific cohorts remains needed. Fourth, evidence on the integration phase of rehabilitation remains limited, constraining recommendations for this critical transition period. Furthermore, the nursing-focused search strategy may have underrepresented relevant literature from rehabilitation medicine that did not explicitly address nursing roles. Finally, the review protocol was retrospectively registered with Open Science Framework Registries rather than prospectively with PROSPERO, as PROSPERO excludes reviews that have commenced data extraction. Prospective registration is preferred because it constrains eligibility before the evidence is seen, and that safeguard is not available here: the protocol was registered on 12 December 2025, after the search had been executed and the synthesis completed, and the registered record was not updated to reflect the procedures actually applied. The registered record and the review as conducted differ in five respects bearing on eligibility, namely the databases searched, the search window, language eligibility, design eligibility and the scope of the population criterion, each reported with its reason in the Methods and in Supplementary Material S3. The registration should therefore be read as documentation of the review’s original plan rather than as evidence that eligibility was fixed before the evidence was seen. The registered exclusion criteria were applied unchanged. The widening of language eligibility did not in the event alter the corpus, since all 51 included reports are English-language publications. The most consequential of these differences is the admission of adjacent-population evidence: 9 of the 51 included studies (17.6%) were conducted in mixed or non-cervical spinal surgical, spinal cord injury, or chronic neck pain populations. This was necessary because several components of postoperative rehabilitation nursing have no evidence base specific to surgically managed cervical spondylotic myelopathy, but it means that part of the synthesis rests on transferable rather than direct evidence, and the corresponding statements should be read as hypotheses for testing in surgically managed cohorts with cervical spondylotic myelopathy rather than as established findings.

### 5.8 Directions for future research

Several areas warrant investigation. Randomized controlled trials are needed to evaluate the effectiveness of nurse-led interventions targeting kinesiophobia in patients with cervical spondylotic myelopathy; the prevalence reported in the included studies, although derived from limited and predominantly single-centre evidence, suggests a target population large enough to warrant quantification in multi-centre work. Prospective studies could validate the proposed Rehabilitation Nursing System Selection Framework in clinical practice. Multi-center studies across different healthcare systems would help establish the generalizability of kinesiophobia prevalence findings. Cost-effectiveness analyses of nurse-led prehabilitation and telerehabilitation programs are needed to inform resource allocation decisions. Development and validation of myelopathy-specific instruments for assessing self-care agency would support standardized assessment and outcome measurement.

## 6 Conclusions

This integrative review synthesised current evidence on postoperative rehabilitation nursing for patients with cervical spondylotic myelopathy and, using Orem's Self-Care Deficit Theory, organised it into four synthesis domains: risk stratification and acute-phase management; phased functional exercise rehabilitation; pain management and psychobehavioural support; and long-term follow-up and telerehabilitation. Its main contribution is an integrated, theory-informed account of the nursing role across the whole postoperative pathway. Because roughly one sixth of the included evidence was drawn from clinically adjacent populations and is transferable rather than myelopathy-specific, this account is offered as a structured basis for evaluation rather than as a definitive summary of established effects. Within this account, psychological barriers (including kinesiophobia and preoperative psychological distress, reported by a limited number of predominantly single-centre studies) are one relevant component that may influence rehabilitation outcomes, and that falls within nursing's scope of practice, rather than the central finding of the review.

The proposed Rehabilitation Nursing System Selection Framework offers a structured, theory-informed approach but is a conceptual framework requiring prospective empirical validation before it can be used as a clinical decision tool. Screening for psychological barriers may be a useful first step to identify patients who could benefit from additional psychological or behavioural support alongside physical rehabilitation.

## Ethical approval

Ethical approval was not required for this integrative review, as it synthesises published literature and involves no primary data collection from human participants.

## Funding

This research did not receive any specific grant from funding agencies in the public, commercial, or not-for-profit sectors.

## CRediT authorship contribution statement

**Xiaoping Wang:** Writing – review & editing, Writing – original draft, Validation, Supervision, Methodology, Data curation. **Xuerong Zhang:** Writing – review & editing, Writing – original draft, Validation, Data curation, Conceptualization. **Qing Zhao:** Validation, Data curation. **Can Liu:** Data curation. **Yanling He:** Data curation. **Meifang Li:** Data curation.

## Declaration of competing interest

The authors declare the following financial interests/personal relationships which may be considered as potential competing interests:

Xiaoping Wang reports that article publishing charges were provided by Zhongshan Xiaolan People’s Hospital (The Fifth People’s Hospital of Zhongshan), Xiaolan Clinical Institute of Shantou University Medical College. The other authors declare that they have no known competing financial interests or personal relationships that could have appeared to influence the work reported in this paper.
