# Supplementary material for: Postoperative functional rehabilitation for cervical spondylotic myelopathy: An integrative review guided by Orem’s self-care deficit theory
Source: Int J Nurs Stud Adv. 2026 Oct 1;11:100696. doi: 10.1016/j.ijnsa.2026.100696 (PMC13671331; doi:10.1016/j.ijnsa.2026.100696)
Supplement: Supplementary file 1 [file mmc1.docx]

**Supplementary Material S1**

**Search Strategy and Database Yields**

**Search date:** February 2025. **Publication window:** 1 January 2014 – 31 January 2025. **Languages:** English and Chinese (applied where supported by the platform).

**Databases searched (5):** PubMed/MEDLINE, Embase (embase.com), CINAHL Complete (EBSCOhost), Web of Science Core Collection, and OpenAlex.

**1. Records identified per database**

| **Database** | **Platform** | **Records identified** |
| --- | --- | --- |
| PubMed/MEDLINE | pubmed.ncbi.nlm.nih.gov | 454 |
| Embase | embase.com (Elsevier) | 779 |
| CINAHL Complete | EBSCOhost | 208 |
| Web of Science | Core Collection (Clarivate) | 833 |
| OpenAlex | api.openalex.org | 2,961 |
| **Total identified** |  | **5,235** |

**Note on reproducibility (Embase).** The figures above are the original counts retrieved in February 2025 and are the counts reported in the PRISMA flow diagram (Figure 2). For the deduplication step, the Embase records were re-exported from embase.com; owing to ongoing database curation and re-indexing, 1,158 records were available at re-export (versus 779 originally retrieved). Because Embase indexes MEDLINE and overlaps substantially with PubMed and Web of Science, this difference has a negligible effect on the number of unique records after deduplication. Deduplication was therefore performed on the records available at export, while the reported identification count retains the original search yield (779).

**2. Deduplication and screening flow**

Records from all databases were combined and deduplicated using a two-step procedure: (i) exact matching on Digital Object Identifier (DOI), followed by (ii) normalised title matching for records without a DOI. The resulting flow was:

| **Stage** | **n** |
| --- | --- |
| Records identified through database searching | 5,235 |
| Duplicate records removed | 1,040 |
| Records screened (title/abstract) | 4,195 |
| Records excluded at title/abstract | 4,008 |
| Reports sought for retrieval | 187 |
| Reports not retrieved (full text unavailable) | 10 |
| Reports assessed for eligibility | 177 |
| Reports excluded, with reasons: | 126 |
| – Not rehabilitation-focused | 58 |
| – Ineligible population | 31 |
| – Outcomes not relevant | 23 |
| – Duplicate report of an included study | 11 |
| – Non-surgical management only | 2 |
| – Trial protocol without outcome data | 1 |
| **Studies included in the review** | **51** |

Arithmetic check: 5,235 − 1,040 = 4,195; 4,195 − 4,008 = 187; 187 − 10 = 177; 177 − 126 = 51; 58 + 31 + 23 + 11 + 2 + 1 = 126. The same figures appear in the main text (Section 3.5) and in Figure 2.

**3. Full search strategies**

**3.1 PubMed/MEDLINE — 454 records**

#1 "Cervical Spondylotic Myelopathy"[MeSH] OR "cervical spondylotic myelopathy"[tiab]

#2 "Cervical myelopathy"[tiab] OR "degenerative cervical myelopathy"[tiab]

#3 "Spinal Cord Compression"[MeSH] AND "Cervical Vertebrae"[MeSH]

#4 #1 OR #2 OR #3

#5 "Decompression, Surgical"[MeSH] OR "Laminoplasty"[MeSH] OR "Diskectomy"[MeSH]

#6 surgery[tiab] OR surgical[tiab] OR operative[tiab] OR postoperative[tiab]

#7 #5 OR #6

#8 "Rehabilitation"[MeSH] OR "Physical Therapy Modalities"[MeSH]

#9 "Rehabilitation Nursing"[MeSH] OR "Nursing Care"[MeSH]

#10 rehabilitation[tiab] OR physiotherapy[tiab] OR "physical therapy"[tiab]

#11 "functional exercise"[tiab] OR "exercise therapy"[tiab] OR mobilization[tiab]

#12 nursing[tiab] OR "nursing care"[tiab] OR "nurse-led"[tiab]

#13 #8 OR #9 OR #10 OR #11 OR #12

#14 #4 AND #7 AND #13

#15 #14 AND ("2014/01/01"[PDAT] : "2025/01/31"[PDAT])

#16 #15 AND (English[Language] OR Chinese[Language]) → 454

**3.2 Embase (embase.com) — 779 records**

#1 ('cervical spondylotic myelopathy':ti,ab,kw OR 'cervical myelopathy':ti,ab,kw OR

'degenerative cervical myelopathy':ti,ab,kw OR 'spinal cord compression'/exp OR

((cervical NEAR/3 myelopath*):ti,ab) OR ((cervical NEAR/3 spondylo*):ti,ab) OR

csm:ti,ab OR dcm:ti,ab) AND ('spine surgery'/exp OR 'decompressive surgery'/exp OR

laminoplasty:ti,ab,kw OR laminectomy:ti,ab,kw OR discectomy:ti,ab,kw OR surg*:ti,ab OR

operat*:ti,ab OR postoperat*:ti,ab OR decompress*:ti,ab OR acdf:ti,ab OR fusion:ti,ab) AND

('rehabilitation'/exp OR 'nursing care'/exp OR 'physical therapy'/exp OR

'exercise therapy'/exp OR 'self care'/exp OR 'patient education'/exp OR rehabilitat*:ti,ab OR

physiotherap*:ti,ab OR ((nurs* NEAR/3 (care OR intervention* OR rehabilitat*)):ti,ab) OR

((functional NEAR/2 exercise*):ti,ab) OR 'self-care':ti,ab OR kinesiophob*:ti,ab OR

((fear NEAR/2 movement):ti,ab)) AND [2014-2025]/py AND ([english]/lim OR [chinese]/lim)	→ 1,050

#2 #1 AND ('article'/it OR 'article in press'/it OR 'clinical trial'/it OR 'review'/it OR

'short survey'/it)	→ 779

**3.3 CINAHL Complete (EBSCOhost) — 208 records**

S1 (MH "Cervical Vertebrae") OR (MH "Spinal Cord Compression")

S2 TI("cervical spondylotic myelopathy" OR "cervical myelopathy" OR

"degenerative cervical myelopathy") OR AB("cervical spondylotic myelopathy")

S3 S1 OR S2

S4 (MH "Surgery, Operative") OR (MH "Spinal Fusion") OR TI(surgery OR surgical OR postoperative)

S5 (MH "Rehabilitation") OR (MH "Rehabilitation Nursing") OR (MH "Physical Therapy")

S6 TI(rehabilitation OR nursing OR "functional exercise" OR physiotherapy)

S7 S5 OR S6

S8 S3 AND S4 AND S7

S9 S8 AND (DT 2014-2025)

S10 S9 AND (LA English OR LA Chinese)	→ 208

**3.4 Web of Science Core Collection — 833 records**

Note: language limits were not applied in the Web of Science search; the topic (TS) search across 2014–2025 retrieved 833 records, which is the count exported and used.

#1 TS=("cervical spondylotic myelopathy" OR "cervical myelopathy" OR

"degenerative cervical myelopathy")

#2 TS=(surgery OR surgical OR decompression OR laminoplasty OR discectomy OR

"anterior cervical")

#3 TS=(rehabilitation OR physiotherapy OR "physical therapy" OR "functional exercise" OR

nursing OR "postoperative care")

#4 #1 AND #2 AND #3

#5 #4 AND PY=(2014-2025)	→ 833

**3.5 OpenAlex (api.openalex.org) — 2,961 records**

#1 title.search:"cervical spondylotic myelopathy" OR title.search:"cervical myelopathy"

OR title.search:"degenerative cervical myelopathy"

#2 #1 AND (abstract.search:rehabilitation OR abstract.search:nursing OR

abstract.search:postoperative OR abstract.search:surgery)

#3 #2 AND publication_year:2014-2025	→ 2,961
