# Supplementary material for: Postoperative functional rehabilitation for cervical spondylotic myelopathy: An integrative review guided by Orem’s self-care deficit theory
Source: Int J Nurs Stud Adv. 2026 Oct 1;11:100696. doi: 10.1016/j.ijnsa.2026.100696 (PMC13671331; doi:10.1016/j.ijnsa.2026.100696)
Supplement: Supplementary file 2 [file mmc2.docx]

**Supplementary Material S2**

**Quality Appraisal Results**

Quality appraisal was conducted using validated tools appropriate for each study design. Randomized controlled trials were assessed using the Joanna Briggs Institute (JBI) Critical Appraisal Checklist for RCTs. Cohort studies (both prospective and retrospective) were evaluated using the JBI Critical Appraisal Checklist for Cohort Studies. Cross-sectional studies were appraised using the JBI Critical Appraisal Checklist for Cross-Sectional Studies. Systematic reviews and meta-analyses were assessed using AMSTAR 2 (A MeaSurement Tool to Assess systematic Reviews 2). Clinical practice guidelines and consensus statements were evaluated using the AGREE II (Appraisal of Guidelines for Research and Evaluation II) instrument. Validation studies were assessed using COSMIN (COnsensus-based Standards for the selection of health Measurement INstruments). Narrative reviews were appraised using SANRA (Scale for the Assessment of Narrative Review Articles). Both tiers of the eligibility framework described in Section 3.4.1 of the main manuscript were appraised with the same design-appropriate instruments; core (Tier 1) and adjacent-population (Tier 2) studies are therefore reported together in the tables below, and Tier 2 studies are identified by a superscript b in Table 1 of the main manuscript. For ease of cross-reference, the appraisal tool and the corresponding overall score or rating for each included study are also summarised in Table 1 of the main manuscript, while the item-level judgments underlying those ratings are provided in the tables below.

## Table S2.1. Quality appraisal of randomized controlled trials (JBI Critical Appraisal Checklist for RCTs)

Y = Yes; N = No; U = Unclear. Q1: Randomization; Q2: Allocation concealment; Q3: Similar groups at baseline; Q4: Participant blinding; Q5: Intervention delivery blinding; Q6: Outcome assessor blinding; Q7: Groups treated identically; Q8: Follow-up complete; Q9: ITT analysis; Q10: Outcomes measured same way; Q11: Outcomes measured reliably; Q12: Appropriate statistics; Q13: Trial design appropriate.

| **Study** | **Q1** | **Q2** | **Q3** | **Q4** | **Q5** | **Q6** | **Q7** | **Q8** | **Q9** | **Q10** | **Q11** | **Q12** | **Q13** | **Score** | **Quality** |
| --- | --- | --- | --- | --- | --- | --- | --- | --- | --- | --- | --- | --- | --- | --- | --- |
| Coronado et al. (2020) | Y | U | Y | N | N | Y | Y | Y | U | Y | Y | Y | Y | 9/13 | Moderate |
| Uehara et al. (2022) | Y | Y | Y | N | N | N | Y | Y | Y | Y | Y | Y | Y | 10/13 | High |

*Note:* The published protocol of a further randomized controlled trial, of virtual-reality–augmented rehabilitation after surgery for cervical spondylotic myelopathy (Wu et al., 2024), was identified at full-text screening but excluded from the review because it reports no outcome data; it was therefore not appraised with the JBI checklist for randomized controlled trials and is not included in this table or in the summary below.

## Table S2.2. Quality appraisal of prospective cohort studies (JBI Critical Appraisal Checklist for Cohort Studies)

Q1: Similar groups; Q2: Similar exposure measurement; Q3: Valid/reliable exposure; Q4: Confounders identified; Q5: Confounders controlled; Q6: Participants free of outcome at start; Q7: Valid/reliable outcome measurement; Q8: Sufficient follow-up; Q9: Follow-up complete/addressed; Q10: Strategies to address incomplete follow-up; Q11: Appropriate statistics.

| **Study** | **Q1** | **Q2** | **Q3** | **Q4** | **Q5** | **Q6** | **Q7** | **Q8** | **Q9** | **Q10** | **Q11** | **Score** | **Quality** |
| --- | --- | --- | --- | --- | --- | --- | --- | --- | --- | --- | --- | --- | --- |
| Akeda et al. (2014) | Y | Y | Y | Y | Y | Y | Y | Y | Y | Y | Y | 11/11 | High |
| Nakashima et al. (2016) | Y | Y | Y | Y | Y | Y | Y | Y | Y | Y | Y | 11/11 | High |
| Nagoshi et al. (2017) | Y | Y | Y | Y | Y | Y | Y | Y | Y | Y | Y | 11/11 | High |
| Tetreault et al. (2015) | Y | Y | Y | Y | Y | Y | Y | Y | Y | Y | Y | 11/11 | High |
| Archer et al. (2014) | Y | Y | Y | Y | Y | Y | Y | Y | Y | Y | Y | 11/11 | High |
| Burgstaller et al. (2017) | Y | Y | Y | Y | Y | Y | Y | Y | Y | Y | Y | 11/11 | High |
| Tetreault et al. (2019) | Y | Y | Y | Y | Y | Y | Y | Y | Y | Y | Y | 11/11 | High |
| Dijkman et al. (2022) | Y | Y | Y | Y | Y | Y | Y | Y | Y | Y | Y | 11/11 | High |
| Nielsen et al. (2014) | Y | Y | Y | Y | Y | N | Y | Y | Y | N | Y | 9/11 | Moderate |
| Haddas et al. (2021a) | Y | Y | Y | Y | Y | N | Y | Y | Y | N | Y | 9/11 | Moderate |
| Haddas et al. (2021b) | Y | Y | Y | Y | Y | N | Y | Y | Y | N | Y | 9/11 | Moderate |

## Table S2.3. Quality appraisal of retrospective cohort studies (JBI Critical Appraisal Checklist for Cohort Studies)

| **Study** | **Q1** | **Q2** | **Q3** | **Q4** | **Q5** | **Q6** | **Q7** | **Q8** | **Q9** | **Q10** | **Q11** | **Score** | **Quality** |
| --- | --- | --- | --- | --- | --- | --- | --- | --- | --- | --- | --- | --- | --- |
| Cox et al. (2014) | Y | Y | Y | Y | Y | Y | Y | Y | Y | Y | Y | 11/11 | High |
| McLynn et al. (2018) | Y | Y | Y | Y | Y | Y | Y | Y | Y | Y | Y | 11/11 | High |
| Zakaria et al. (2019) | Y | Y | Y | Y | Y | Y | Y | Y | Y | Y | Y | 11/11 | High |
| Nagoshi et al. (2019) | Y | Y | Y | Y | Y | Y | Y | Y | Y | Y | Y | 11/11 | High |
| Sherrod et al. (2023) | Y | Y | Y | Y | Y | Y | Y | Y | Y | Y | Y | 11/11 | High |
| Chan et al. (2023) | Y | Y | Y | Y | Y | Y | Y | Y | Y | Y | Y | 11/11 | High |
| Ren et al. (2017) | Y | Y | Y | Y | Y | N | Y | Y | N | Y | Y | 9/11 | Moderate |
| Bui et al. (2021) | Y | Y | Y | Y | Y | N | Y | Y | N | Y | Y | 9/11 | Moderate |
| Lorentz et al. (2023) | Y | Y | Y | Y | Y | N | Y | Y | N | Y | Y | 9/11 | Moderate |
| Passias et al. (2021) | Y | Y | Y | Y | Y | N | Y | Y | N | Y | Y | 9/11 | Moderate |
| Catz et al. (2024) | Y | Y | Y | Y | Y | N | Y | Y | N | Y | Y | 9/11 | Moderate |
| Wang et al. (2023) | Y | Y | Y | Y | Y | N | Y | Y | N | Y | Y | 9/11 | Moderate |
| Rahman et al. (2024) | Y | Y | Y | Y | Y | N | Y | Y | N | Y | Y | 9/11 | Moderate |

## Table S2.4. Quality appraisal of cross-sectional studies (JBI Critical Appraisal Checklist)

Q1: Inclusion criteria clearly defined; Q2: Study subjects and setting described; Q3: Valid/reliable exposure measurement; Q4: Objective, standard criteria used; Q5: Confounders identified; Q6: Confounders controlled; Q7: Valid/reliable outcome measurement; Q8: Appropriate statistics.

| **Study** | **Q1** | **Q2** | **Q3** | **Q4** | **Q5** | **Q6** | **Q7** | **Q8** | **Score** | **Quality** |
| --- | --- | --- | --- | --- | --- | --- | --- | --- | --- | --- |
| Oh et al. (2017) | Y | Y | Y | Y | Y | Y | N | Y | 7/8 | Moderate |
| Zhong et al. (2023) | Y | Y | Y | Y | Y | Y | N | Y | 7/8 | Moderate |
| Chen et al. (2024) | Y | Y | Y | Y | Y | Y | N | Y | 7/8 | Moderate |

## Table S2.5. Quality appraisal of systematic reviews and meta-analyses (AMSTAR 2)

AMSTAR 2 overall confidence ratings: High = No or one non-critical weakness; Moderate = More than one non-critical weakness; Low = One critical flaw with or without non-critical weaknesses; Critically Low = More than one critical flaw with or without non-critical weaknesses.

| **Study** | **Overall confidence** | **Critical weaknesses** | **Non-critical weaknesses** |
| --- | --- | --- | --- |
| Gómara-Toldrà et al. (2014) | High | None | 1 |
| Badran et al. (2018) | High | None | 1 |
| Valenza-Peña et al. (2024) | High | None | 0 |
| Zhou et al. (2023) | High | None | 0 |

## Table S2.6. Quality appraisal of clinical practice guidelines and consensus statements (AGREE II)

D1 = Scope and Purpose; D2 = Stakeholder Involvement; D3 = Rigour of Development; D4 = Clarity of Presentation; D5 = Applicability; D6 = Editorial Independence. Scores represent scaled domain percentages.

| **Study** | **D1** | **D2** | **D3** | **D4** | **D5** | **D6** | **Overall** |
| --- | --- | --- | --- | --- | --- | --- | --- |
| Fehlings et al. (2017) | 95% | 88% | 92% | 96% | 85% | 92% | High |
| Davies et al. (2022) | 88% | 80% | 85% | 90% | 75% | 88% | High |

## Table S2.7. Quality appraisal of other study designs

COSMIN = COnsensus-based Standards for the selection of health Measurement INstruments; SANRA = Scale for the Assessment of Narrative Review Articles; JBI = Joanna Briggs Institute.

| **Study** | **Design** | **Appraisal tool** | **Score** | **Quality** |
| --- | --- | --- | --- | --- |
| Kopjar et al. (2015) | Validation study | COSMIN | Adequate | High |
| Kato et al. (2015) | Multicenter validation | COSMIN | Adequate | High |
| Tetreault et al. (2017) | Validation study | COSMIN | Adequate | High |
| Kalsi-Ryan et al. (2020) | Validation study | COSMIN | Adequate | High |
| Cheng et al. (2020) | Quasi-experimental | JBI Quasi-exp | 7/9 | Moderate |
| Nouri et al. (2015) | Narrative review | SANRA | 8/12 | Moderate |
| Badhiwala et al. (2020) | Narrative review | SANRA | 10/12 | High |
| Choi and Kang (2020) | Narrative review | SANRA | 8/12 | Moderate |
| Jannelli et al. (2020) | Narrative review | SANRA | 8/12 | Moderate |
| Boerger et al. (2022) | Narrative review | SANRA | 10/12 | High |
| Nouri et al. (2022) | Narrative review | SANRA | 10/12 | High |
| Gibson et al. (2018) | Narrative review | SANRA | 8/12 | Moderate |
| McCormick et al. (2020) | Narrative review | SANRA | 8/12 | Moderate |
| Hejrati et al. (2022) | Narrative review | SANRA | 8/12 | Moderate |
| Huang et al. (2024) | Narrative review | SANRA | 8/12 | Moderate |
| Lee et al. (2024) | Narrative review | SANRA | 8/12 | Moderate |

# Summary of Quality Appraisal

Of the 51 included studies, 28 (54.9%) were rated as high quality, 23 (45.1%) as moderate quality, and 0 (0%) as low quality. The most common methodological limitations were related to blinding in intervention studies and incomplete control of confounding variables in observational studies.

**Quality distribution by study design:**

| **Study design** | **High** | **Moderate** | **Low** | **Total** |
| --- | --- | --- | --- | --- |
| RCT | 1 | 1 | 0 | 2 |
| Prospective cohort | 8 | 3 | 0 | 11 |
| Retrospective cohort | 6 | 7 | 0 | 13 |
| Cross-sectional | 0 | 3 | 0 | 3 |
| Systematic review/MA | 4 | 0 | 0 | 4 |
| Guideline/Consensus | 2 | 0 | 0 | 2 |
| Validation study | 4 | 0 | 0 | 4 |
| Narrative review | 3 | 8 | 0 | 11 |
| Other (quasi-experimental) | 0 | 1 | 0 | 1 |
| **Total** | **28 (54.9%)** | **23 (45.1%)** | **0 (0%)** | **51 (100%)** |
