# Supplementary material for: Postoperative functional rehabilitation for cervical spondylotic myelopathy: An integrative review guided by Orem’s self-care deficit theory
Source: Int J Nurs Stud Adv. 2026 Oct 1;11:100696. doi: 10.1016/j.ijnsa.2026.100696 (PMC13671331; doi:10.1016/j.ijnsa.2026.100696)
Supplement: Supplementary file 3 [file mmc3.docx]

**Supplementary Material S3**

**Protocol registration and departures from the registered record**

The protocol for this review was registered with the Open Science Framework (OSF) Registries (https://osf.io/4jh7q) on 12 December 2025. Registration was retrospective: it followed both the execution of the search in February 2025 and the completion of the synthesis, and it was made at that point because PROSPERO does not accept reviews that have progressed to data extraction. The registered record reproduces the protocol as originally drafted for this review and was not updated before posting to reflect the procedures that had by then been applied. Table S3.1 sets out, for each element of the registered protocol, what the registered record states, what was applied in the review as conducted, and the reason for any difference; elements that do not differ are listed as well, so that the correspondence can be verified in full. The registered record and the review as conducted differ in six respects bearing on the search and the inclusion criteria, and in one respect bearing on the appraisal instruments. The exclusion criteria were applied as registered. None of the procedures reported here was adopted in response to the findings of an individual study, and none altered the review question or the outcomes of interest. Two further differences concern the strength of claims rather than method, and in both the claim was narrowed rather than widened during peer review. The eligibility criteria reported in the eligibility criteria of the main manuscript are those finally applied. Table S3.2 lists the nine studies admitted under the adjacent-evidence tier and the reason for admitting each.

**Table S3.1. The registered record, the procedure applied, and the reason for each difference**

| **Protocol parameter** | **As recorded in the OSF registration** | **As applied in the review reported here** | **Difference from the registered record, and the reason** |
| --- | --- | --- | --- |
| Date of registration | 12 December 2025 (open-ended registration; the record shows the same date for creation and registration) | Not applicable | Registration was retrospective and followed both execution of the search in February 2025 and completion of the synthesis. It was made at that point because PROSPERO does not accept reviews that have progressed to data extraction. The registered record reproduces the protocol as originally drafted for this review and was not updated before posting to reflect the procedures that had by then been applied. Because registration followed the synthesis, it provides no protection against selective reporting; this is stated among the limitations in the main manuscript. |
| Title | Postoperative Rehabilitation Nursing and Functional Exercise for Cervical Spondylotic Myelopathy: An Orem Theory-Guided Integrative Review | Postoperative Functional Rehabilitation for Cervical Spondylotic Myelopathy: An Integrative Review Guided by Orem’s Self-Care Deficit Theory | Editorial refinement during peer review. The population, question, and scope are unchanged. |
| Objectives 1 and 2 | Synthesise current evidence on postoperative rehabilitation nursing and functional exercise for cervical spondylotic myelopathy; operationalise Orem’s Self-Care Deficit Nursing Theory into a clinically actionable nursing framework | Unchanged in substance. The framework is presented as a conceptual framework requiring prospective empirical validation before use as a clinical decision tool | Claim narrowed during peer review. The framework has not been prospectively validated, so presenting it as clinically actionable would have overstated its status. |
| Objective 3 | Identify the psychobehavioural burden (kinesiophobia and psychological distress) as a critical barrier to rehabilitation success | Psychobehavioural findings are reported as one component of the synthesis rather than as its central finding; the third objective is stated as delineating research gaps and priorities | Claim narrowed during peer review. The prevalence estimates derive from limited, predominantly single-centre evidence and do not support the registered framing. |
| Review design | Integrative review following Whittemore and Knafl (2005), reported per PRISMA 2020 | Unchanged | No difference |
| Conceptual framework | Orem’s Self-Care Deficit Nursing Theory as an a priori analytical framework; the three nursing systems guide evidence synthesis | Unchanged | No difference |
| Data sources | PubMed/MEDLINE; CINAHL Complete (via EBSCOhost); Web of Science Core Collection; Google Scholar; reference-list screening | PubMed/MEDLINE; CINAHL Complete; Web of Science Core Collection; Embase; OpenAlex; reference-list screening | Difference 1. Google Scholar was replaced by OpenAlex and Embase was added. Google Scholar does not return exportable, stably ordered result sets and so cannot support a reproducible and deduplicable search record, whereas OpenAlex returns structured, identifier-indexed records that can be queried and deduplicated reproducibly. Embase was added for coverage of European clinical and rehabilitation literature that is incompletely indexed elsewhere. Reference-list screening was retained as registered. Both changes were made when the search was re-run in February 2025. |
| Search window | January 2014 to October 2024 | January 2014 to January 2025 | Difference 2. The start date is as registered. The initial search of November 2024 ran to the registered end date; when the search was re-run in February 2025 the end date was moved forward so that the window ran to the month preceding execution. Seminal work published before 2014 is cited for theoretical and clinical context but is not counted among the included studies. |
| Date of search execution | November 2024, per the registered timeline | Initial search November 2024; search re-run in February 2025 across the amended set of databases and the extended window. The review reports the re-run | The initial search prompted the changes to the database set and the search window recorded above. During extraction it became apparent that the registered database set would not support a reproducible, deduplicable search record and did not cover part of the literature required, and that the registered window had closed. The search was therefore re-executed in full rather than updated piecemeal, so that the review would rest on a single, fully documented search. Screening was carried out afresh on the complete yield of the re-run, and Figure 2 reports the February 2025 search. |
| Language eligibility | Non-English publications excluded | English or Chinese | Difference 3. Chinese was added so that reports of postoperative rehabilitation nursing published in Chinese could be considered. In the event no Chinese-language report met the remaining criteria; all 54 included studies are English-language publications, so this amendment did not alter the corpus. |
| Population and eligibility scope | Adults aged 18 years or over with cervical spondylotic myelopathy who had undergone surgical decompression | Two-tier inclusion. Tier 1 (core evidence, n = 42): as registered, together with cervical spine surgical cohorts in which myelopathy was an operative indication. Tier 2 (adjacent, transferable evidence, n = 9): admitted only where all three conditions stated in the eligibility criteria were met | Difference 4. Several components of postoperative rehabilitation nursing had no evidence base specific to surgically managed cervical spondylotic myelopathy. Rather than leave those components unsupported or drop them from the synthesis, a defined tier of adjacent-population evidence was admitted under three explicit conditions, appraised with the same instruments, labelled in Table 1, and never used as the sole basis for a practice-oriented statement. The extension is confined to adjacent surgical and musculoskeletal populations; it does not extend to the non-surgical management of cervical myelopathy, which remains excluded. The nine Tier 2 studies and the reason for admitting each are listed in Table S3.2. |
| Exclusion criteria | Non-surgical management only; traumatic or neoplastic myelopathy; conference abstracts or case reports with fewer than five patients | Applied as registered, unchanged | No difference. |
| Eligible study designs | Randomized controlled trials; quasi-experimental studies; cohort studies; systematic reviews; clinical guidelines; expert consensus statements | As registered, plus cross-sectional studies, ambispective cohort studies, meta-analyses, measurement and validation studies, and narrative reviews | Difference 5. Design eligibility was broadened in keeping with integrative-review methodology, which admits diverse forms of evidence provided that the role of each in the synthesis is stated. Measurement studies were required in order to establish the instruments used to grade myelopathy severity and track recovery; narrative reviews were admitted as contextual rather than practice-determining evidence. Each study is labelled by evidence type in Table 1. |
| Outcomes of interest | Neurological recovery (mJOA), functional independence, quality of life, pain, complications, psychological outcomes, length of stay | Unchanged | No difference |
| Quality appraisal | Joanna Briggs Institute critical appraisal tools; AMSTAR 2 for systematic reviews; AGREE II for clinical guidelines | As registered, plus COSMIN for measurement and validation studies and SANRA for narrative reviews | Difference 6, consequent on Difference 5. The registered instruments do not cover measurement studies or narrative reviews, both of which became eligible. Item-level judgments for every included study are reported in Supplementary Material S2. |
| Screening and selection | Not specified in the registered record | Title and abstract, then full-text screening, by two independent reviewers, with disagreement resolved by discussion | Not a difference in procedure. The registered record did not specify a screening procedure; the procedure applied is reported in the study selection section of the main manuscript. |
| Data synthesis | Narrative synthesis following Popay et al. (2006); thematic content analysis informed by Braun and Clarke (2006); theory-driven mapping onto Orem’s constructs | Unchanged in method; the thematic analysis citation was updated to the current edition (Braun and Clarke, 2023) | No difference in method. Citation updated only. |
| Ethics | No ethical approval required, as the review synthesises published literature and involves no primary data collection | Unchanged | No difference |

**Table S3.2. The nine studies admitted as adjacent (Tier 2) evidence, with the reason for admission**

| **Study** | **Population studied** | **Reason for admission as Tier 2 evidence** | **Where it is used in the synthesis, and how it is qualified** |
| --- | --- | --- | --- |
| Akeda et al. (2014) | Adults undergoing mixed spinal surgery | No Tier 1 study evaluated an institutional venous thromboembolism prophylaxis protocol | Domain 1, postoperative surveillance and complication prevention. The text states that this evidence derives from mixed spinal-surgical rather than myelopathy-specific cohorts |
| Cox et al. (2014) | Adults undergoing spine surgery | No Tier 1 study evaluated early multimodal prophylaxis combined with early mobilisation | Domain 1, postoperative surveillance and complication prevention, cited collectively with Table 1 under the same qualification |
| Nielsen et al. (2014) | Adults after spine surgery | No Tier 1 study audited postoperative pain management | Domain 3, multimodal pain management. Framed as evidence of a care gap, not of a myelopathy-specific effect |
| Archer et al. (2014) | Adults after lumbar and cervical degenerative spine surgery | No Tier 1 study followed patients prospectively from early postoperative kinesiophobia to later pain, disability and physical health | Domain 3, kinesiophobia and psychological distress. Explicitly identified as an adjacent spinal-surgical population |
| Gómara-Toldrà et al. (2014) | Adults with spinal cord injury | The one Tier 1 systematic review of post-surgical rehabilitation (Badran et al., 2018) identified a single eligible trial and could not support conclusions; no Tier 1 study addressed participation-oriented rehabilitation goals | Domain 2, evidence base for rehabilitation, where the text already describes the goals as transferable to myelopathy |
| Burgstaller et al. (2017) | Adults after lumbar spinal stenosis surgery | No Tier 1 study distinguished persistent from baseline fear-avoidance beliefs | Domain 3, kinesiophobia and psychological distress. Explicitly identified as an adjacent spinal-surgical population |
| McLynn et al. (2018) | Adults undergoing elective spine surgery | Provides the general spine-surgery denominator against which the cervical-specific venous thromboembolism rates reported by Tier 1 registry studies are interpreted | Domain 1, postoperative surveillance and complication prevention, cited collectively with Table 1 under the same qualification |
| Haddas et al. (2021b) | Adults with degenerative spine pathology | No Tier 1 study derived thresholds relating kinesiophobia and demoralization to gait and balance dysfunction | Domain 3, kinesiophobia and psychological distress. Described as complementary to the myelopathy-specific finding of Haddas et al. (2021a), which is a Tier 1 study |
| Valenza-Peña et al. (2024) | Adults with chronic neck pain | No Tier 1 study evaluated telerehabilitation | Domain 4, long-term follow-up and telerehabilitation. Stated as a transferable rather than myelopathy-specific delivery model |

**Note.** Tier 2 studies were appraised with the same design-appropriate instruments as Tier 1 studies and appear in the same appraisal tables in Supplementary Material S2. They are marked with a superscript b in Table 1 of the main manuscript. No practice-oriented statement in the Results rests on a Tier 2 study alone. AGREE II, Appraisal of Guidelines for Research and Evaluation II; AMSTAR 2, A MeaSurement Tool to Assess systematic Reviews 2; COSMIN, COnsensus-based Standards for the selection of health Measurement INstruments; JBI, Joanna Briggs Institute; OSF, Open Science Framework; SANRA, Scale for the Assessment of Narrative Review Articles.
